# Adjuvant chemoradiotherapy versus radiotherapy alone in women with high-risk endometrial cancer (PORTEC-3): patterns of recurrence and post-hoc survival analysis of a randomised phase 3 trial

**DOI:** 10.1016/S1470-2045(19)30395-X

**Published:** 2019-09

**Authors:** Stephanie M de Boer, Melanie E Powell, Linda Mileshkin, Dionyssios Katsaros, Paul Bessette, Christine Haie-Meder, Petronella B Ottevanger, Jonathan A Ledermann, Pearly Khaw, Romerai D'Amico, Anthony Fyles, Marie-Helene Baron, Ina M Jürgenliemk-Schulz, Henry C Kitchener, Hans W Nijman, Godfrey Wilson, Susan Brooks, Sergio Gribaudo, Diane Provencher, Chantal Hanzen, Roy F Kruitwagen, Vincent T H B M Smit, Naveena Singh, Viet Do, Andrea Lissoni, Remi A Nout, Amanda Feeney, Karen W Verhoeven-Adema, Hein Putter, Carien L Creutzberg, PORTEC Study Group, M McCormack, K Whitmarsh, R Allerton, D Gregory, P Symonds, PJ Hoskin, M Adusumalli, A Anand, R Wade, A Stewart, W Taylor, LCHW Lutgens, H Hollema, E Pras, A Snyers, GH Westerveld, JJ Jobsen, A Slot, JM Mens, TC Stam, B Van Triest, EM Van der Steen-Banasik, KAJ De Winter, MA Quinn, I Kolodziej, J Pyman, C Johnson, A Capp, R Fossati, A Colombo, S Carinelli, A Ferrero, G Artioli, C Davidson, CM McLachlin, P Ghatage, PVC Rittenberg, L Souhami, G Thomas, P Duvillard, D Berton-Rigaud, N Tubiana-Mathieu

**Affiliations:** a Department of Radiation Oncology, Leiden University Medical Center, Leiden, Netherlands; b Department of Pathology, Leiden University Medical Center, Leiden, Netherlands; c Department of Medical Statistics, Leiden University Medical Center, Leiden, Netherlands; d Department of Clinical Oncology, Barts Health NHS Trust, London, UK; e Department of Cellular Pathology, Barts Health NHS Trust, London, UK; f Division of Cancer Medicine, Peter MacCallum Cancer Centre, Melbourne, VIC, Australia; g Division of Radiation Oncology, Peter MacCallum Cancer Centre, Melbourne, VIC, Australia; h Department of Surgical Sciences, Gynecologic Oncology, Città della Salute and S Anna Hospital, University of Turin, Turin, Italy; i Canadian Cancer Trials Group, Department of Obstetrics and Gynaecology, University of Sherbrooke, Sherbrooke, QC, Canada; j Department of Radiotherapy, Institut Gustave Roussy, Villejuif, France; k Department of Medical Oncology, Radboudumc, Nijmegen, Netherlands; l Cancer Research UK, London, UK; m UCL Cancer Trials Centre, UCL Cancer Institute, London, UK; n Division of Radiation Oncology, ASST-Lecco, Ospedale AManzoni, Lecco, Italy; o Canadian Cancer Trials Group, Radiation Medicine Program, Princess Margaret Cancer Centre, Toronto, ON, Canada; p Department of Radiotherapy, Centre Hospitalier Régional Universitaire de Besançon, Besançon, France; q Department of Radiation Oncology, University Medical Center Utrecht, Netherlands; r Institute of Cancer Sciences, University of Manchester, Manchester, UK; s Department of Gynaecologic Oncology, University Medical Center Groningen, University of Groningen, Groningen, Netherlands; t Department of Pathology, Central Manchester Hospitals NHS Foundation Trust, Manchester Royal Infirmary, Manchester, UK; u Department of Radiation Oncology, Auckland City Hospital, Auckland, New Zealand; v Department of Oncology – Radiotherapy, A.O.U. Città della Salute e della Scienza di Torino, Turin, Italy; w Department of Gynaecologic Oncology, Hôpital Notre-Dame de Montreal, Montreal, QC, Canada; x Department of Radiation Oncology, Centre Henri Becquerel, Rouen, France; y Department of Obstetrics and Gynaecology, Maastricht University Medical Centre, Maastricht, Netherlands; z GROW - School for Oncology and Developmental Biology, Maastricht, Netherlands; aa Department of Radiation Oncology, Liverpool Cancer Therapy Centre, Liverpool, NSW, Australia; ab Gynecologic Oncology Unit, Department of Obstetrics and Gynecology, San Gerardo Hospital, University of Milano-Bicocca, Monza, Italy; ac Comprehensive Cancer Center Netherlands, Rotterdam, Netherlands

## Abstract

**Background:**

The PORTEC-3 trial investigated the benefit of combined adjuvant chemotherapy and radiotherapy versus pelvic radiotherapy alone for women with high-risk endometrial cancer. We updated the analysis to investigate patterns of recurrence and did a post-hoc survival analysis.

**Methods:**

In the multicentre randomised phase 3 PORTEC-3 trial, women with high-risk endometrial cancer were eligible if they had International Federation of Gynaecology and Obstetrics (FIGO) 2009 stage I, endometrioid grade 3 cancer with deep myometrial invasion or lymphovascular space invasion, or both; stage II or III disease; or stage I–III disease with serous or clear cell histology; were aged 18 years and older; and had a WHO performance status of 0–2. Participants were randomly assigned (1:1) to receive radiotherapy alone (48·6 Gy in 1·8 Gy fractions given on 5 days per week) or chemoradiotherapy (two cycles of cisplatin 50 mg/m^2^ given intravenously during radiotherapy, followed by four cycles of carboplatin AUC5 and paclitaxel 175 mg/m^2^ given intravenously), by use of a biased coin minimisation procedure with stratification for participating centre, lymphadenectomy, stage, and histological type. The co-primary endpoints were overall survival and failure-free survival. Secondary endpoints of vaginal, pelvic, and distant recurrence were analysed according to the first site of recurrence. Survival endpoints were analysed by intention-to-treat, and adjusted for stratification factors. Competing risk methods were used for failure-free survival and recurrence. We did a post-hoc analysis to analyse patterns of recurrence with 1 additional year of follow-up. The study was closed on Dec 20, 2013; follow-up is ongoing. This study is registered with ISRCTN, number ISRCTN14387080, and ClinicalTrials.gov, number NCT00411138.

**Findings:**

Between Nov 23, 2006, and Dec 20, 2013, 686 women were enrolled, of whom 660 were eligible and evaluable (330 in the chemoradiotherapy group, and 330 in the radiotherapy-alone group). At a median follow-up of 72·6 months (IQR 59·9–85·6), 5-year overall survival was 81·4% (95% CI 77·2–85·8) with chemoradiotherapy versus 76·1% (71·6–80·9) with radiotherapy alone (adjusted hazard ratio [HR] 0·70 [95% CI 0·51–0·97], p=0·034), and 5-year failure-free survival was 76·5% (95% CI 71·5–80·7) versus 69·1% (63·8–73·8; HR 0·70 [0·52–0·94], p=0·016). Distant metastases were the first site of recurrence in most patients with a relapse, occurring in 78 of 330 women (5-year probability 21·4%; 95% CI 17·3–26·3) in the chemoradiotherapy group versus 98 of 330 (5-year probability 29·1%; 24·4–34·3) in the radiotherapy-alone group (HR 0·74 [95% CI 0·55–0·99]; p=0·047). Isolated vaginal recurrence was the first site of recurrence in one patient (0·3%; 95% CI 0·0–2·1) in both groups (HR 0·99 [95% CI 0·06–15·90]; p=0·99), and isolated pelvic recurrence was the first site of recurrence in three women (0·9% [95% CI 0·3–2·8]) in the chemoradiotherapy group versus four (0·9% [95% CI 0·3–2·8]) in the radiotherapy-alone group (HR 0·75 [95% CI 0·17–3·33]; p=0·71). At 5 years, only one grade 4 adverse event (ileus or obstruction) was reported (in the chemoradiotherapy group). At 5 years, reported grade 3 adverse events did not differ significantly between the two groups, occurring in 16 (8%) of 201 women in the chemoradiotherapy group versus ten (5%) of 187 in the radiotherapy-alone group (p=0·24). The most common grade 3 adverse event was hypertension (in four [2%] women in both groups). At 5 years, grade 2 or worse adverse events were reported in 76 (38%) of 201 women in the chemoradiotherapy group versus 43 (23%) of 187 in the radiotherapy-alone group (p=0·002). Sensory neuropathy persisted more often after chemoradiotherapy than after radiotherapy alone, with 5-year rates of grade 2 or worse neuropathy of 6% (13 of 201 women) versus 0% (0 of 187). No treatment-related deaths were reported.

**Interpretation:**

This updated analysis shows significantly improved overall survival and failure-free survival with chemoradiotherapy versus radiotherapy alone. This treatment schedule should be discussed and recommended, especially for women with stage III or serous cancers, or both, as part of shared decision making between doctors and patients. Follow-up is ongoing to evaluate long-term survival.

**Funding:**

Dutch Cancer Society, Cancer Research UK, National Health and Medical Research Council, Project Grant, Cancer Australia Grant, Italian Medicines Agency, and the Canadian Cancer Society Research Institute.

## Introduction

Women with endometrial cancer generally have a favourable prognosis;^1^ only about 15–20% have high-risk disease characteristics with an increased incidence of distant metastases and cancer-related death.^2^, ^3^, ^4^ High-risk endometrial cancer is defined as endometrioid endometrial cancer stage I, grade 3 with deep invasion, stage II or III endometrioid endometrial cancer (no residual disease), or non-endometrioid (serous or clear cell) histology.^1^

Women with high-risk endometrial cancer have been treated with pelvic external-beam radiotherapy for several decades. Findings of clinical trials comparing adjuvant chemotherapy alone with external-beam radiotherapy alone have shown no differences in survival outcomes.^5^, ^6^ Because a higher incidence of pelvic relapses has been reported with chemotherapy alone compared with a treatment schedule including external-beam radiotherapy,^7^, ^8^ the combination of external-beam radiotherapy with chemotherapy has been explored in clinical trials.

**Research in context**

**Evidence before this study**

We searched PubMed for clinical studies published in English between Jan 1, 1980, and Dec 31, 2006, with the terms “endometrial cancer” AND “radiation therapy” AND “chemotherapy” AND “survival” OR “failure free survival” AND with the terms “endometrial cancer” AND “serous” AND “radiation therapy” AND “chemotherapy” AND “survival” OR “failure free survival”. Six relevant publications were identified. Among these, three randomised trials compared adjuvant chemotherapy with radiotherapy. No differences in overall and progression-free survival were found, whereas an increased frequency of pelvic relapse was reported after chemotherapy alone. Therefore, after these results, the next step was to explore the combination of concurrent chemotherapy and radiotherapy. Since the start of recruitment to the PORTEC-3 trial in 2006, the results of a pooled analysis of the NSGO-EC-9501/EORTC-55991 trial and the ManGO ILIADE-III trial were published, showing a significant improvement in progression-free survival and non-significant improvement in overall survival in women treated with four cycles of platinum-based chemotherapy given sequentially before or after pelvic radiotherapy versus those treated with radiotherapy alone. Results of two relevant trials (Gynecologic Oncology Group [GOG]-GOG 249 and GOG-258) have been published recently. The GOG-249 trial did not show improved progression-free survival with three cycles of carboplatin–paclitaxel with vaginal brachytherapy compared with pelvic radiotherapy in women with stage I–II disease with high (intermediate) risk factors. In the GOG-258 trial, women with stage III–IV endometrial cancer were randomly assigned to receive chemoradiotherapy (the same schedule as that used in the PORTEC-3 trial) or six cycles of carboplatin–paclitaxel alone. No differences in overall or recurrence-free survival were reported, but significantly more vaginal and pelvic or para-aortic recurrences were reported in the chemotherapy group than in the chemoradiotherapy group. The added value of chemotherapy to radiotherapy alone for women with serous cancer has, to our knowledge, been reported in retrospective series only. In a large retrospective study done in 135 women with stage I–IVA serous endometrial cancer, recurrence-free survival was improved by the combination of radiotherapy and chemotherapy. Subgroup analyses in randomised trials have not confirmed such benefits for serous cancers. The NSGO/EORTC trial reported improved progression-free survival with the addition of chemotherapy to radiotherapy for endometrioid cancers, but no such benefit was found for serous cancers.

**Added value of this study**

We report on the patterns of recurrence and provide updated survival outcomes of patients with high-risk endometrial cancer treated in the international randomised phase 3 PORTEC-3 trial. Patients were randomly assigned to receive pelvic radiotherapy alone or the combination of radiotherapy with concurrent (two cycles of cisplatin) and adjuvant (four cycles of carboplatin–paclitaxel) chemotherapy. Both overall and failure-free survival were significantly improved in women treated with combined adjuvant chemotherapy and radiotherapy compared with those treated with radiotherapy alone. In our post-hoc analysis of survival outcomes, the greatest absolute benefit was found for women with stage III or serous cancers, or both. In women with a recurrence, the majority had distant metastases. Pelvic control was excellent in both groups.

**Implications of all the available evidence**

Combined adjuvant chemotherapy and radiotherapy should be discussed and recommended as a new standard of care, especially for women with stage III endometrial cancer or serous cancers, or both. Shared decision making between doctors and their patients remains essential to weigh the costs and benefits for individual patients.

The randomised phase 3 PORTEC-3 trial was initiated to investigate the benefit of a combined adjuvant chemotherapy and radiotherapy (chemoradiotherapy) schedule compared with external-beam radiotherapy alone for women with high-risk endometrial cancer. Previously reported efficacy results with a time-based analysis and a median follow-up of 60·2 months (IQR 48·1–73·1) showed a significant 7% improvement in failure-free survival for patients treated with chemoradiotherapy compared with those treated with radiotherapy alone (76% *vs* 69% at 5 years) without a significant difference in overall survival (82% *vs* 77% at 5 years).^9^ In a subgroup analysis, the largest failure-free survival benefit with chemoradiotherapy (11%; 69% *vs* 58% at 5 years) was observed in women with stage III disease, who have a higher baseline risk of recurrence than women with stage I–II disease.

The aim of the present analysis of the PORTEC-3 trial is to present the patterns of recurrence, treatment, and survival after recurrence and an updated (post-hoc) analysis of the primary endpoints with prolonged follow-up.

## Methods

### Study design and participants

PORTEC-3 was an open-label, multicentre, randomised intergroup phase 3 trial led by the Dutch Gynaecological Oncology Group (DGOG). The trial was done at 103 centres (oncology centres, university hospitals, regional hospitals, or radiation oncology centres with referrals from regional hospitals) in six clinical trial groups collaborating in the Gynaecological Cancer Intergroup. Participating groups were the National Cancer Research Institute (NCRI; UK), the Australia and New Zealand Gynaecologic Oncology Group (ANZGOG; Australia and New Zealand), the Mario Negri Gynaecologic Oncology Group (MaNGO; Italy), the Canadian Cancer Trials Group (Canadian Cancer Trials Group; Canada), and Fedegyn (France).

Details about patient selection and treatment have been published previously.^9^, ^10^ All patients first underwent surgery. In brief, surgery consisted of total abdominal or laparoscopic hysterectomy and bilateral salpingo-oophorectomy. The extent of lymph node removal was left to the discretion of the participating centres, although pelvic lymph node debulking and para-aortic lymph node sampling were recommended in case of macroscopic positive pelvic nodes or para-aortic nodes, or both. Full surgical staging (including omentectomy, peritoneal biopsies, and lymph node sampling) was recommended for patients with serous or clear cell cancer. FIGO 2009 staging was assigned on the basis of surgical and pathological findings. Central pathology review was mandatory before randomisation to confirm eligibility for study entry.^11^

Eligible patients were women with histologically confirmed endometrioid endometrial cancer with either International Federation of Gynecology and Obstetrics (FIGO) 2009 stage IA grade 3 with documented lymphovascular space invasion; stage IB grade 3 disease; stage II disease; stage IIIA, IIIB (parametrial invasion), or IIIC disease; or stage IA–III with serous or clear cell histology (IA with invasion). Eligibility criteria also included WHO performance score 0–2; adequate bone marrow function (white blood cell count ≥3·0 × 10^9^ cells per L, platelets ≥100 × 10^9^ per L), liver function (bilirubin ≤1·5 × upper limit of normal, aspartate aminotransferase to alanine aminotransferase ratio ≤2·5 × upper limit of normal) and kidney function (creatinine clearance >60 mL/min calculated according to Cockroft and Gault or >50 mL/min edetic acid [EDTA] clearance), and age 18 years or older (without an upper age limit, because elderly women might benefit from the study treatment if they were deemed fit enough to undergo chemotherapy). Exclusion criteria were uterine sarcoma or carcinosarcoma, previous malignancy (except for non-melanomatous skin cancer) within the past 10 years, previous pelvic radiotherapy, previous hormonal therapy or chemotherapy, bulky cervical involvement with radical hysterectomy, inflammatory bowel disease, residual macroscopic tumour, impaired renal or cardiac function, neuropathy grade 2 or worse, hearing impairment grade 3 or worse, or a congenital hearing disorder.

Written informed consent was obtained from all patients. The protocol was approved by the Dutch Cancer Society and by the ethics committees of all participating groups. The study protocol is available online.

### Randomisation and masking

Patients were randomly assigned (1:1) to open-label treatment groups with chemoradiotherapy or radiotherapy alone, by use of a biased-coin minimisation procedure ensuring balance overall and within each stratum of the stratification factors (participating centre, lymphadenectomy [yes *vs* no], FIGO 2009 stage of cancer [IA *vs* IB *vs* II *vs* III]), and histological type [endometrioid carcinoma *vs* serous or clear cell carcinoma]). Patients were registered and randomly assigned by the data centres of the participating groups and treatment was assigned with a web-based application. Participants, physicians, and investigators were not masked to treatment allocation.

### Procedures

External-beam pelvic radiotherapy was given to patients in both treatment groups to a total dose of 48·6 Gy in 1·8 Gy fractions, 5 days per week. Specifications of the treatment schedule have been reported previously.^9^, ^10^, ^11^ In case of glandular or stromal cervical involvement, or both, a brachytherapy boost was given. Brachytherapy dose was equivalent to 14 Gy in 2 Gy fractions (with a recommended scheme of 10 Gy high-dose rate in fractions of 5 Gy). Treatment was recommended to start within 4–6 weeks of surgery, but no later than 8 weeks after surgery. Overall radiotherapy treatment time was not to exceed 50 days.

In the chemoradiotherapy group, women received two cycles of cisplatin 50 mg/m^2^ administered intravenously in the first and fourth week of external-beam radiotherapy, followed by four cycles of carboplatin AUC5 and paclitaxel 175 mg/m^2^ administered intravenously at 21-day intervals. This schedule was based on the RTOG-9708 trial,^12^ with substitution of cisplatin by carboplatin in the adjuvant phase. Adjuvant chemotherapy was started within 3 weeks after completion of external-beam pelvic radiotherapy, and with a 28-day interval from the second concurrent cycle. Cisplatin was postponed for 1 week in the event of haematological, renal, or other toxicities or discontinued if recovery required more than 1 week or in the case of grade 2 or worse neuropathy. Carboplatin was postponed or stopped in case of severe haematological toxicity. Paclitaxel was postponed if grade 2 neuropathy developed and stopped if recovery exceeded 1 week or if grade 3 neuropathy developed. Details about chemotherapy stopping rules have been described previously.^10^

Follow-up was focused on patient history and pelvic examination to detect adverse events (grade ≥2 according to Common Terminology Criteria for Adverse Events [CTCAE] version 3.0) and symptoms of recurrent disease, with annual chest radiographs, blood counts, and chemistry tests (including CA-125) until 5 years after randomisation. Long-term follow-up of vital status and events was required at 7 years and 10 years. Health-related quality-of-life assessments were done at baseline, at the end of radiotherapy, at 6-month intervals from randomisation until 24 months, and at 36 and 60 months.

Patients who immediately withdrew informed consent after randomisation without any information were excluded from the analysis, as were patients who were ineligible for the study. There were no other criteria for a patient to be removed from the study.

### Outcomes

The co-primary endpoints were overall survival and failure-free survival. Overall survival was defined as the time from randomisation to death from any cause. Failure-free survival was defined as the time from randomisation to any relapse, or death related to endometrial cancer or treatment, whichever occurred first. Women who were alive were censored at the date of their last follow-up. Secondary endpoints were vaginal, pelvic, or distant recurrence; treatment-related toxicity; and health-related quality of life.^10^ Recurrences were analysed according to first site of recurrence as well as the total number of recurrences. Simultaneous vaginal and pelvic recurrence was considered pelvic recurrence; and simultaneous vaginal, pelvic, and distant recurrence was considered distant recurrence. Abdominal recurrences outside the pelvic area (peritoneal carcinomatosis, liver, and para-aortic lymph nodal metastases) were considered distant metastases, with specification of site. After diagnosis of any relapse, treatment information was required and follow-up continued according to protocol guidelines. An extensive update on quality of life will be reported separately in a future publication.

### Statistical analysis

The PORTEC-3 trial was powered (80%) to detect a 10% difference in 5-year overall survival between the treatment groups (increase from 65% to 75%; hazard ratio [HR] 0·67), with a two-sided test at an α level of 0·05. 198 overall survival events were required, with a minimum of 655 patients. A prespecified interim analysis was done in September, 2013, after 48 overall survival events (a third of the required events) had occurred. The final analysis of the co-primary endpoints was published in February, 2018, with permission of the data and safety monitoring board as a time-based analysis rather than an event-based analysis since a median follow-up of 60·2 months had been reached and because the event rate was lower than expected.

To maintain an overall α of 0·05 with a nominal α level for the interim analysis of 0·0002, the final analysis was done with a nominal α of 0·0498. The final time-based analysis was done with a correlation of 0·7859 between the test statistics of the co-primary endpoints overall survival and failure-free survival (based on 136 overall survival events and 186 failure-free survival events), and a nominal α of 0·0309 was used for each of the analyses, resulting in an overall α level of 0·0498.^13^ The sequential rejection principle^14^ implies that if the null hypothesis of no treatment difference for one of the co-primary endpoints is rejected (p<0·0309), the null hypothesis of no treatment difference for the other co-primary endpoint can then be assessed at the 0·05 level, while still retaining a family-wise error rate of 0·05.

The current analysis was done to evaluate patterns of recurrences, together with a non-prespecified post-hoc analysis of survival after recurrence as well as an updated analysis of overall survival and failure-free survival with prolonged follow-up and 5-year adverse events. Final database lock was on Nov 29, 2018.

We did statistical analyses using SPSS, version 23.0.0.2, and R, version 3.5.1. All analyses were done by intention to treat, excluding ineligible patients and those who immediately withdrew informed consent after randomisation. Differences in relapse and survival rates between the groups were tested with the log-rank test and Cox regression analysis. The analysis of the primary endpoints was adjusted for the stratification factors (participating group, lymphadenectomy, stage of cancer, and histological type), since a stratified minimisation procedure was used at randomisation.^15^, ^16^ For the adjusted analysis, stratification factors were included as covariates in the Cox model. The proportional hazards assumption for treatment was checked by use of Schoenfeld residuals for overall survival and failure-free survival, and was not found to be violated.^17^ Patient characteristics were compared with a χ^2^ test. Survival after recurrence was compared with a log-rank test and for this analysis the first site of recurrence was used. Competing risk methods were used for analysis of failure-free survival and recurrence, by calculating cumulative incidences and Fine-Gray regression.^18^ For failure-free survival, intercurrent death was used as a competing risk. For the first failure analysis of recurrences, all other recurrences and death were used as competing risks. Median survival after recurrence was calculated as the first timepoint at which the Kaplan-Meier curve was below 50% survival. The IQR was calculated similarly. In the multivariable analysis, the following covariates were included together with treatment: stage, histological type and grade, type of surgery, participating groups, lymphovascular space invasion, and age. The median follow-up and IQR was estimated with the reverse Kaplan-Meier method.

This study is registered with ISRCTN, number ISRCTN14387080, and ClinicalTrials.gov, number NCT00411138.

### Role of the funding source

The funders of the study had no role in study design, data collection, data interpretation, data analysis, or writing of this report. The central data manager (KWV), the chief investigator (CLC), the associated investigators (SMdB and RAN), and the trial statistician (HP) had full access to all the data. The decision to submit for publication was made after discussion within the trial management group. The corresponding author and chief investigator had full access to all the data in the study and had final responsibility for the decision to submit for publication.

## Results

**Figure 1:**
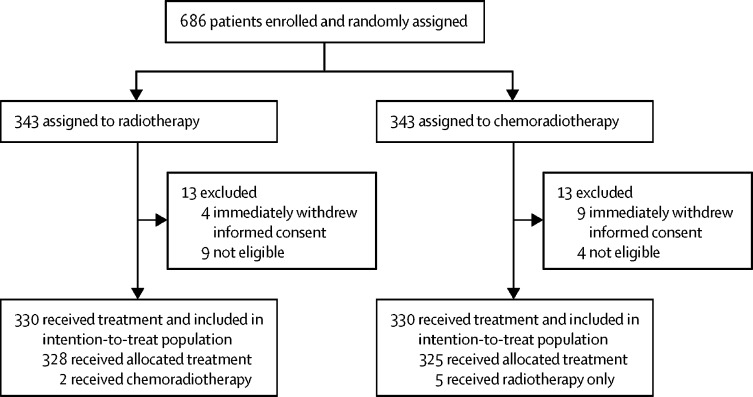
Trial profile

Between Nov 23, 2006, and Dec 20, 2013, 686 women were enrolled and randomly assigned to chemoradiotherapy (n=343) or radiotherapy (n=343); 26 patients were excluded after randomisation (figure 1), resulting in 660 patients in the intention-to-treat analysis (330 in each group). Median follow-up was 72·6 months (IQR 59·9–85·6) at the time of the current analysis and 75% of participants had reached at least 5 years of follow-up.

Patient characteristics were well balanced between the treatment groups (table 1). The median age of the enrolled patients was 62 years (IQR 56–68). Baseline characteristics of patients with and without a recurrence are given in table 1.

**Table 1:** Baseline characteristics, by treatment group and by recurrence status

		**Chemoradiotherapy group (n=330)**	**Radiotherapy alone group (n=330)**	**Any recurrence (n=185)**	**No recurrence (n=475)**	p value*
Age at randomisation, years						
	Median (IQR)	62·4 (56·5–67·9)	62·0 (55·8–68·2)	63·9 (59·0–69·9)	60·9 (55·5–67·6)	··
Age group						<0·0001
	<60 years	128 (39%)	140 (42%)	54 (29%)	214 (45%)	··
	60–69 years	144 (44%)	128 (39%)	86 (47%)	186 (39%)	··
	≥70 years	58 (18%)	62 (19%)	45 (24%)	75 (16%)	··
FIGO 2009 stage						<0·0001
	Stage IA	39 (12%)	39 (12%)	14 (8%)	64 (14%)	··
	Stage IB	59 (18%)	58 (18%)	22 (12%)	95 (20%)	··
	Stage II	80 (24%)	90 (27%)	42 (23%)	128 (27%)	··
	Stage IIIA	46 (14%)	37 (11%)	22 (12%)	61 (13%)	··
	Stage IIIB	18 (6%)	24 (7%)	19 (10%)	23 (5%)	··
	Stage IIIC	88 (27%)	82 (25%)	66 (36%)	104 (22%)	··
Histological grade and type						<0·0001
	Endometrioid endometrial carcinoma† grade 1–2	127 (39%)	131 (40%)	61 (33%)	197 (42%)	··
	Endometrioid endometrial carcinoma† grade 3	107 (32%)	106 (32%)	59 (32%)	154 (32%)	··
	Serous cancer	53 (16%)	52 (16%)	47 (25%)	58 (12%)	··
	Clear cell cancer	29 (9%)	33 (10%)	17 (9%)	45 (10%)	··
	Other	14 (4%)	8 (2%)	1 (1%)	21 (4%)	··
Myometrial invasion						0·010
	<50%	116 (35%)	123 (37%)	53 (29%)	186 (39%)	··
	≥50%	212 (65%)	206 (63%)	132 (71%)	286 (61%)	··
	Missing	2	1	0	3	··
Lymphovascular space invasion						0·008
	Present	197 (60%)	192 (58%)	124 (67%)	265 (56%)	··
	Absent	133 (40%)	138 (42%)	61 (33%)	210 (44%)	··
WHO performance status score						0·70
	0–1	323 (98%)	324 (98%)	181 (98%)	466 (99%)	··
	≥2	5 (2%)	5 (2%)	4 (2%)	6 (1%)	··
	Missing	2	1	0	3	··
Type of surgery						
	TAH and BSO	97 (29%)	97 (29%)	58 (31%)	136 (29%)	0·866
	TAH and BSO plus LND or full staging	141 (43%)	132 (40%)	72 (39%)	201 (42%)	··
	TLH and BSO	45 (14%)	42 (13%)	25 (14%)	62 (13%)	··
	TLH and BSO plus LND or full staging	47 (14%)	59 (18%)	30 (16%)	76 (16%)	··

Data are n (%) or median (IQR), unless otherwise stated. FIGO=International Federation of Gynaecology and Obstetrics. TAH=total abdominal hysterectomy. BSO=bilateral salpingo-oopherectomy. LND=lymph node dissection. TLH=total laparoscopic hysterectomy. Missing values are not taken into account for the percentages.

* χ^2^ test of variables between patients with versus without any recurrence.

† Endometrioid endometrial carcinoma including mixed tumours with less than 25% of serous or clear cell component.

Radiotherapy was completed in 329 (>99%) of 330 women in the chemoradiotherapy group and in 325 (99%) of 330 in the radiotherapy alone group. Vaginal brachytherapy was given to 309 (47%) of 660 patients: 151 (46%) of 330 patients on chemoradiotherapy and 158 (48%) of 330 on radiotherapy alone. In the chemoradiotherapy group, concurrent cisplatin was completed by 304 (92%) of 330 women, adjuvant carboplatin by 262 (79%), and adjuvant paclitaxel by 233 (71%). At least one dose reduction of cisplatin (to 40 mg/m^2^) was recorded for five (2%) patients, of carboplatin (from AUC5 to AUC4) for 36 (11%) patients, and of paclitaxel (from 175 mg/m^2^ to 135 mg/m^2^) for 50 (15%) patients. Chemotherapy was discontinued in 61 (18%) of 330 patients: because of toxicity in 31 (9%), the patient's decision in 20 (6%), disease progression in seven (2%), and for other reasons in three (1%).^9^, ^10^

At the final database lock (Nov 29, 2018), 150 patients had died (65 in the chemoradiotherapy group and 85 in the radiotherapy alone group) and 189 patients had a failure-free survival event (84 in the chemoradiotherapy group and 105 in the radiotherapy alone group). This number of events comprised 76% (150 of 198) of required overall survival events and 95% (189 of 198) of failure-free survival events for the final analysis. Most deaths were related to endometrial cancer: 53 (82%) of 65 in the chemoradiotherapy group versus 76 (89%) of 85 in the radiotherapy group. Among women assigned to chemoradiotherapy, other causes of death were second cancers (in five [8%] patients), other intercurrent disease (in three [5%] patients), and complications of treatment for metastatic disease (in two [3%] patients). Among women assigned to radiotherapy alone, other causes of death were second cancers (in five [6%] patients), other intercurrent disease (in one [1%] patient), and complications of treatment for metastatic disease (in one [1%] patient). The cause of death was uncertain for two patients treated with chemoradiotherapy and two patients treated with radiotherapy alone; these deaths were considered failure-free survival events since a relation to endometrial cancer or to treatment for endometrial cancer could not be excluded.^9^

**Figure 2:**
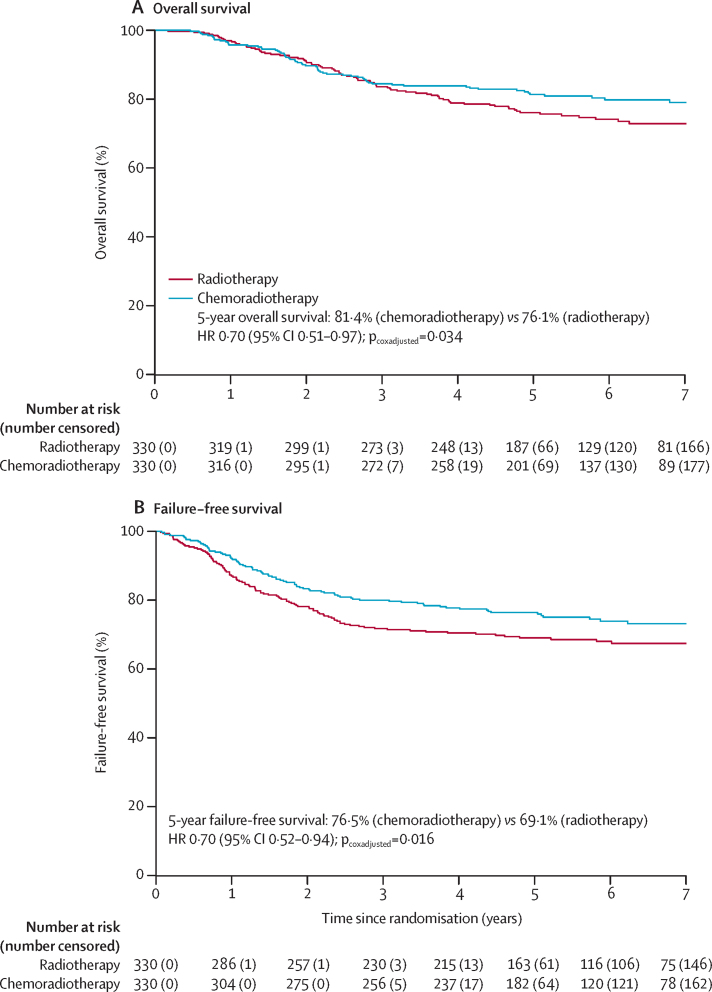
Kaplan-Meier survival curves for overall survival (A) and failure-free survival (B) in all patients HR=hazard ratio.

Estimated 5-year overall survival adjusted for stratification factors was 81·4% (95% CI 77·2–85·8) with chemoradiotherapy versus 76·1% (71·6–80·9) with radiotherapy alone (HR 0·70 [95% CI 0·51–0·97], p=0·034). Estimated 5-year failure-free survival adjusted for stratification factors was 76·5% (95% CI 71·5–80·7) with chemoradiotherapy versus 69·1% (63·8–73·8) with radiotherapy alone (HR 0·70 [95% CI 0·52–0·94], p=0·016; figure 2).

**Figure 3:**
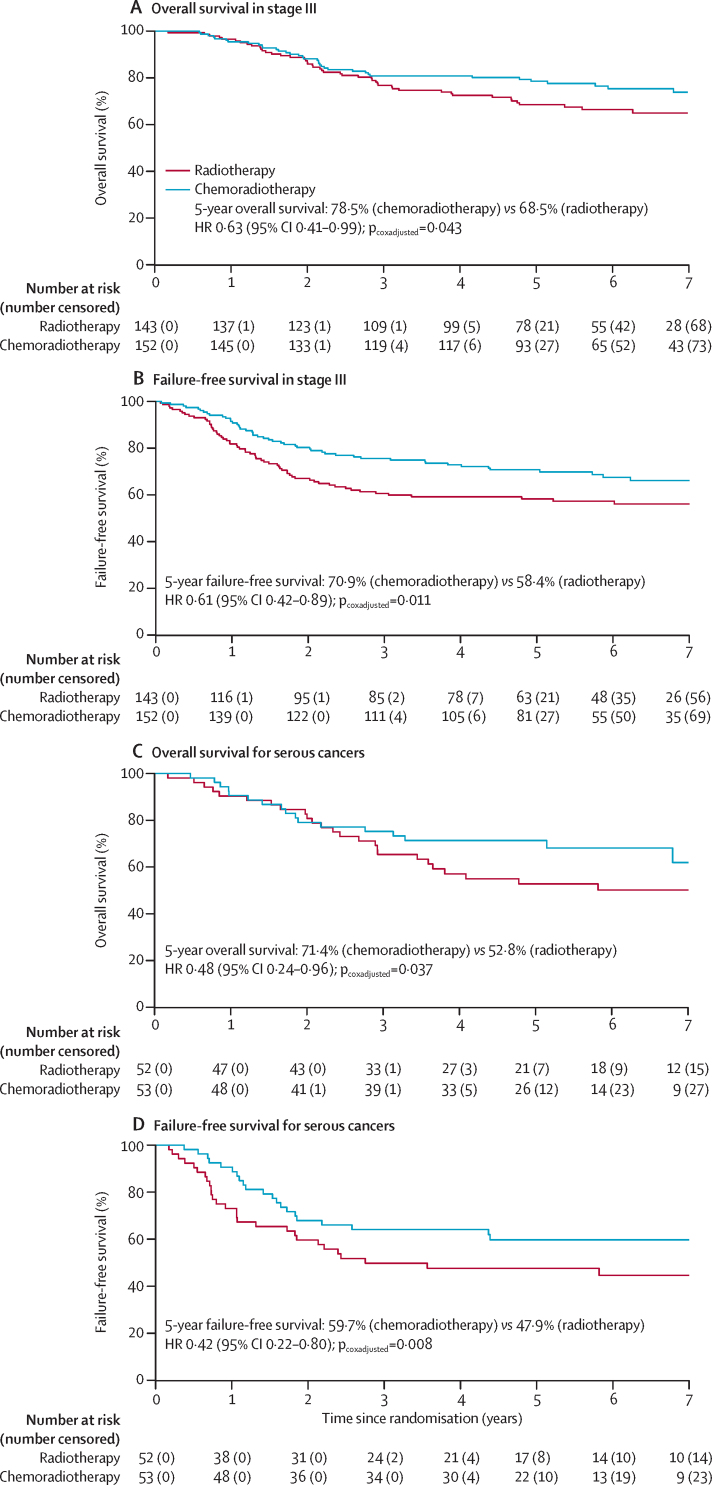
Kaplan-Meier survival curves for overall survival (A) and failure-free survival (B) among patients with stage III endometrial cancer, and overall survival (C) and failure-free survival (D) for patients with serous cancer HR=hazard ratio.

In our post-hoc exploratory analysis of survival outcomes by disease stage, women with stage III disease had significantly lower overall survival and failure-free survival than women with stage I–II disease, irrespective of treatment received (appendix p 6). In women with stage III endometrial cancer, 5-year overall survival was 78·5% (95% CI 72·2–85·4) with chemoradiotherapy versus 68·5% (61·2–76·7) with radiotherapy alone (HR 0·63 [95% CI 0·41–0·99]; p=0·043), and 5-year failure-free survival was 70·9% (95% CI 62·9–77·4) with chemoradiotherapy versus 58·4% (49·8–66·0) with radiotherapy alone (HR 0·61 [95% CI 0·42–0·89]; p=0·011; figure 3A, B). In women with stage I–II disease, 5-year overall survival was 83·8% (95% CI 78·4–89·5) with chemoradiotherapy versus 82·0% (95% CI 76·5–87·7) with radiotherapy alone HR 0·84 [95% CI 0·52–1·38]; p=0·50), and 5-year failure-free survival was 81·3% (95% CI 74·7–86·3) with chemoradiotherapy versus 77·3% (95% CI 70·5–82·7) with radiotherapy alone (HR 0·87 [95% CI 0·56–1·36]; p=0·54; appendix p 8).

When comparing serous cancers with all other histologies in a post-hoc exploratory subgroup analysis, women with serous cancers had significantly lower overall survival and failure-free survival than did those with other histologies irrespective of treatment received (appendix p 7), and the difference in overall survival and failure-free survival among the different disease stages was more pronounced for serous cancers than for other histologies (appendix pp 10, 11). After adjusting for stratification factors, significant improvements in overall survival and failure-free survival were observed for serous cancers treated with chemoradiotherapy versus radiotherapy alone: 5-year overall survival was 71·4% (95% CI 60·1–84·7) with chemoradiotherapy versus 52·8% (40·6–68·6) with radiotherapy alone (HR 0·48 [95% CI 0·24–0·96]; p=0·037), and 5-year failure-free survival was 59·7% (95% CI 45·1–71·6) with chemotherapy versus 47·9% (33·9–60·6) with radiotherapy alone (HR 0·42 [95% CI 0·22–0·80]; p=0·008; figure 3C, 3D).

Stage, histological type and grade, type of surgery, participating groups, lymphovascular space invasion, and age were included in the prespecified multivariable analysis for overall and failure-free survival. Compared with radiotherapy alone, chemoradiotherapy significantly improved overall and failure-free survival in the presence of these factors (appendix p 5). Most factors, except for lymphadenectomy (and lymphovascular space invasion for overall survival) were correlated with overall survival and failure- free survival (appendix p 5). In a post-hoc analysis of survival outcomes by type of surgery, the type of surgery (laparotomy *vs* laparoscopy) did not affect overall survival or failure-free survival (appendix p 13).

Distant metastases were the first type of recurrence in most patients with a recurrence, and occurred in 98 of 330 patients (5-year probability 29·1% [95% CI 24·4–34·3] in the radiotherapy-only group compared with 78 of 330 patients (5-year probability 21·4% [17·3–26·3] in the chemoradiotherapy group (HR 0·74 [95% CI 0·55–0·99]; p=0·047; table 2). In the radiotherapy group, 74 (76%) of these 98 recurrences were distant recurrence only (of which 12 were isolated para-aortic nodal recurrences); 20 patients had combined distant and pelvic recurrences (including two with distant, pelvic, and vaginal recurrences), and four had combined distant and vaginal recurrences. In the chemoradiotherapy group, 63 (81%) of 78 distant recurrences were distant only (of which nine were isolated para-aortic nodal recurrences); 11 were combined distant and pelvic recurrences (including one with distant, pelvic, and vaginal recurrence), and four were combined distant and vaginal recurrences.

**Table 2:** Recurrence outcomes by treatment group

	**Number of events**	**5-year probability (95% CI)**	**Hazard ratio (95% CI)**	Log-rank p value*
**Vaginal recurrence (first recurrence)**				
Chemoradiotherapy	1	0·3% (0·0–2·1)	0·99 (0·06–15·90)	0·99
Radiotherapy alone	1	0·3% (0·0–2·1)	··	··
**Pelvic recurrence (first recurrence)**				
Chemoradiotherapy	3	0·9% (0·3–2·8)	0·75 (0·17–3·33)	0·71
Radiotherapy alone	4	0·9% (0·3–2·8)	··	··
**Distant metastases (first recurrence)**				
Chemoradiotherapy	78	21·4% (17·3–26·3)	0·74 (0·55–0·99)	0·047
Radiotherapy alone	98	29·1% (24·4–34·3)	··	··
**Vaginal recurrence (total)**				
Chemoradiotherapy	8	2·1% (1·0–4·4)	0·99 (0·37–2·65)	0·99
Radiotherapy alone	8	2·1% (1·0–4·4)	··	··
**Pelvic recurrence (total)**				
Chemoradiotherapy	20	5·5% (3·5–8·6)	0·63 (0·36–1·11)	0·11
Radiotherapy alone	31	8·5% (5·9–12·1)	··	··
**Distant metastases (total)**				
Chemoradiotherapy	80	22·1% (17·9–27·0)	0·75 (0·56–1·01)	0·057
Radiotherapy alone	99	29·4% (24·7–34·6)	··	··

* Unadjusted for stratification factors.

Isolated vaginal recurrence was the first site of recurrence in one patient (0·3% [95% CI 0·0–2·1]) in both groups (HR 0·99 [95% CI 0·06–15·90]; p=0·99), and isolated pelvic recurrence was the first site of recurrence in four women (0·9% [0·3–2·8]) in the radiotherapy group and in three (0·9% [0·3–2·8]) in the chemoradiotherapy group (HR 0·75 [95% CI 0·17–3·33]; p=0·71; table 2).

**Figure 4:**
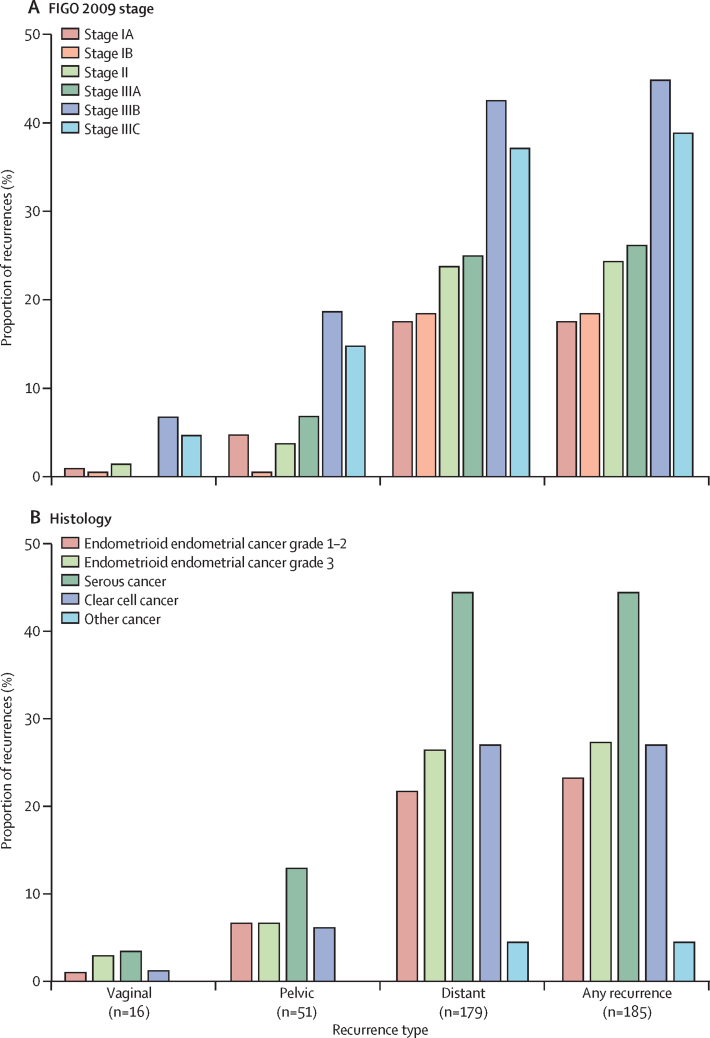
Recurrences divided by stage (A) and histology (B) FIGO=International Federation of Gynaecology and Obstetrics.

Figure 4 shows recurrences by cancer stage and histological type. Recurrences were highest in women with stage IIIB (19 [45·2%] of 42) and stage IIIC (66 [38·8%] of 170) disease. Across the different histological types, the greatest risk of recurrence was for serous cancers (47 [44·8%] of 105), followed by clear cell cancers (17 [27·4%] of 62) and grade 3 endometrioid endometrial cancer (59 [27·7%] of 213).

Median survival after recurrence was 1·4 years (IQR 0·5–3·7) in the total PORTEC-3 cohort and did not differ significantly between the two treatment groups: 1·4 years (0·7–3·7) for patients in the radiotherapy group and 1·2 years (0·4–8·9) in the chemoradiotherapy group (HR 1·06 [95% CI 0·75–1·51]; p=0·72; table 3). In both groups, about 9–10% of patients (n=33; 16 in the radiotherapy group and 17 in the chemoradiotherapy group) survived for several years after recurrence (appendix p 12). In these cases, recurrences were more often solitary metastases and low grade in type, and more often treated with hormonal therapy (data not shown).

**Table 3:** Median survival after recurrence

	**Number of events**	Median survival,*years (IQR)	**Hazard ratio (95% CI)**	**Log-rank p value**†
**Vaginal or pelvic recurrence**				
Chemoradiotherapy	4	1·2 (0·7–NR)	··	··
Radiotherapy alone	6	1·4 (0·9–NR)	··	··
Total	10	1·4 (0·9–NR)	1·31 (0·26–6·55)	0·74
**Distant metastases**				
Chemoradiotherapy	76	1·2 (0·3–8·9)	··	··
Radiotherapy alone	93	1·5 (0·7–3·7)	··	··
Total	169	1·4 (0·5–3·7)	1·05 (0·73–1·50)	0·79
**Any recurrence**				
Chemoradiotherapy	82	1·2 (0·4–8·9)	··	··
Radiotherapy alone	103	1·4 (0·7–3·7)	··	··
Total	185	1·4 (0·5–3·7)	1·06 (0·75–1·51)	0·72

NR=not reached.

* For median survival after recurrence, the first site of recurrence was used.

† Unadjusted for stratification factors.

In terms of treatment after recurrence, patients in the radiotherapy group were more likely to be treated with chemotherapy for their first recurrence than those in the chemoradiotherapy group (48 [47%] of 103 women *vs* 30 [37%] of 82 women), and most women in the radiotherapy group (43 [90%] of 48) received carboplatin plus paclitaxel (details of treatment received by the other five women are provided in the appendix (p 3). In the chemoradiotherapy group, various combinations of chemotherapy agents were used (data not shown) and only 12 (40%) of 30 patients received carboplatin plus paclitaxel again (details of treatment received by the other 18 women are provided in the appendix p 3). An overview of all treatment modalities is given in the appendix (pp 3, 14).

A comprehensive overview of adverse events during treatment and the first few years after treatment has been provided in previous publications.^9^, ^10^ After longer follow-up, we found no significant differences between the two treatment groups in grade 3 or worse adverse events at 12, 36, and 60 months after randomisation (appendix p 4). 60 months after randomisation, grade 3 adverse events were reported for 16 (8%) of 201 women in the chemoradiotherapy group versus ten (5%) of 187 women in the radiotherapy group (p=0·24). Only one grade 4 adverse event (ileus or obstruction) was reported (in the chemoradiotherapy group). The most frequently reported grade 3 adverse events were hypertension (in four women [2%] in both groups), any pain (in three [1%] women in the chemoradiotherapy group *vs* three [2%] in the radiotherapy group) and other toxicities (in three [1%] women in the chemoradiotherapy group *vs* two [1%] in the radiotherapy group).

At 60 months, grade 2 or worse adverse events were reported for 76 (38%) of 201 women in the chemoradiotherapy group versus 43 (23%) of 187 women in the radiotherapy group (p=0·002). Sensory neuropathy grade 2 or worse was the major difference between the two groups at 5 years, seen in 13 (6%) of 201 patients after chemoradiotherapy versus no patients in the radiotherapy group (table 4).

**Table 4:** Grade 2 and 3 adverse events reported at 60 months after randomisation

		**Grade 2**			Grade 3*		
		Chemoradiotherapy group (n=201)	Radiotherapy alone group (n=187)	p value†	Chemoradiotherapy group (n=201)	Radiotherapy alone group (n=187)	p value‡
Any		59 (29%)	33 (18%)	0·002	16 (8%)	10 (5%)	0·24
	Allergic reaction or hypersensitivity	0	0	0·48	0	1 (1%)	0·48
	Auditory or hearing	4 (2%)	1 (1%)	0·29	2 (1%)	1 (1%)	1·00
	Hypertension	16 (8%)	16 (9%)	0·87	4 (2%)	4 (2%)	1·00
	Lymphatics (oedema)	5 (2%)	0	0·06	0	0	1·00
Gastrointestinal (any)		16 (8%)	9 (5%)	0·19	2 (1%)	1 (1%)	1·00
	Constipation	3 (1%)	1 (1%)	1·00	0	1 (1%)	1·00
	Diarrhoea	7 (3%)	7 (4%)	1·00	0	0	1·00
	Ileus or obstruction	2 (1%)	1 (1%)	0·37	2 (1%)	0	0·50
Haematological (any)		5 (2%)	5 (3%)	1·00	0	0	1·00
	Lymphocytes	3 (1%)	4 (2%)	0·74	0	0	1·00
Infection (without neutropenia)		2 (1%)	0	0·12	2 (1%)	0	0·50
Neuropathy (any)		13 (6%)	0	<0·0001	1 (<1%)	0	1·01
	Motor neuropathy	1 (<1%)	0	0·50	1 (<1%)	0	1·02
	Sensory neuropathy	12 (6%)	0	<0·0001	1 (<1%)	0	1·03
Neurology (other)		1 (<1%)	0	0·25	2 (1%)	0	0·50
Pain (any)		13 (6%)	5 (3%)	0·15	3 (1%)	3 (2%)	1·00
	Joint pain	7 (3%)	2 (1%)	0·14	2 (1%)	1 (1%)	1·00
	Muscle pain	1 (<1%)	1 (1%)	0·61	0	1 (1%)	0·48
	Back/pelvic/limbs	0	2 (1%)	0·11	0	1 (1%)	0·48
	Abdomen/cramps	2 (1%)	0	0·12	2 (1%)	0	0·50
	Other	2 (1%)	1 (1%)	1·00	1 (<1%)	1 (1%)	1·00
Musculoskeletal (other)		0	1 (1%)	1·00	1 (<1%)	0	1·00
Genitourinary		··	··	··	··	··	··
	Incontinence	8 (4%)	9 (5%)	1·00	0	0	1·00
	Urinary frequency	8 (4%)	2 (1%)	0·14	0	1 (1%)	0·48
Constitutional		··	··	··	··	··	··
	Fatigue	0	3 (2%)	0·11	0	0	1·00
	Other	0	0	1·00	1 (<1%)	0	1·00
Other toxicity		4 (2%)	4 (2%)	1·00	3 (1%)	2 (1%)	1·00

Data are n (%).

* Only one grade 4 adverse event was reported 60 months after randomisation for a women treated in the chemoradiotherapy group (ileus or obstruction).

† Significance level: p<0·01 for grade ≥2 events.

‡ Significance level: p<0·01 for grade ≥3 events. The prevalence of toxicity graded according to the Common Terminology Criteria for Adverse Events version 3.0 was calculated at each timepoint. For each adverse event, the maximum grade per patient was recorded (worst ever by patient).

## Discussion

These updated results of the PORTEC-3 trial, with a longer median follow-up of 72 months and with 75% of participants having reached 5 years of follow-up, showed a significant improvement in both overall and failure-free survival with chemoradiotherapy versus radiotherapy alone for high-risk endometrial cancer. The absolute improvement at 5 years was 5% (HR 0·70; 95% CI 0·51–0·97) for overall survival and 7% (0·70; 0·52–0·94) for failure-free survival. Most recurrences were at distant sites, with excellent local and regional control in both groups. Women in the radiotherapy group were more likely to receive chemotherapy for their first recurrence. Women with serous cancers had worse overall survival and failure-free survival than those with other histological types, and for these women a significant improvement of overall survival (absolute improvement 19%; HR 0·48 [95% CI 0·24–0·96]) and failure-free survival (absolute improvement 12%; 0·42 [0·22–0·80]) was found with chemoradiotherapy compared with radiotherapy alone.

External-beam radiotherapy has been the standard adjuvant treatment for women with high-risk endometrial cancer for several decades. Since trials comparing adjuvant chemotherapy alone with external-beam radiotherapy alone did not show differences in survival,^5^, ^6^ the combination of external-beam radiotherapy with four cycles of platinum-based chemotherapy was investigated in the randomised NSGO-EC-9501/EORTC-55991 trial.^19^ A pooled analysis with the ManGO ILIADE-III trial showed a significant improvement in progression-free survival at 5 years with chemoradiotherapy versus radiotherapy alone (78% *vs* 69%, p=0·01) and a non-significant improvement in 5-year overall survival (82% *vs* 75%; p=0·07).^19^ The RTOG 9708 phase 2 trial investigated combined external-beam radiotherapy with concurrent and adjuvant chemotherapy, with both treatment modalities started early after surgery. This combination showed manageable toxicity and a 5-year overall survival of 85% and relapse-free survival of 79%. The PORTEC-3 trial confirmed these high survival rates in a large randomised trial, with estimated 5-year overall survival of 79% and 5-year failure-free survival of 73% in the chemoradiotherapy group.

High-risk endometrial cancer comprises a heterogeneous group of tumours with both early-stage disease with high-risk features and advanced stage endometrial cancer and non-endometrioid tumours. When analysing results by stage, women with stage III endometrial cancer had a significantly higher risk of recurrence than did those with stage I–II disease. For women with stage I–II endometrial cancer, combined adjuvant treatment yielded only a small absolute improvement of 2% (HR 0·84; 95% CI 0·52–1·38) in 5-year overall survival and of 4% (0·87; 0·56–1·36) in failure-free survival. These results are supported by the results of the Gynecologic Oncology Group (GOG)-249 trial, in which women with stage I–II endometrial cancer were randomly assigned to receive external-beam radiotherapy or vaginal brachytherapy and three cycles of carboplatin–paclitaxel chemotherapy. No differences in overall survival or recurrence-free survival were reported, but pelvic and para-aortic recurrences were significantly more frequent after vaginal brachytherapy and chemotherapy.^20^ Taking the results of the GOG-249 and PORTEC-3 trials together, a minimal benefit in failure-free survival and overall survival in stage I–II endometrial cancer achieved with chemoradiotherapy does not seem to justify the increased frequency of adverse events, impaired quality of life, and longer treatment duration of combined treatment for these patients.

For women with stage III endometrial cancer in PORTEC-3, a significant improvement in overall survival (HR 0·63) and in failure-free survival (HR 0·61) was found with chemoradiotherapy versus radiotherapy alone. In the GOG-258 trial, 813 women with stage III–IVA endometrial cancer were randomly assigned to receive pelvic external-beam radiotherapy with concurrent and adjuvant chemotherapy (with the same schedule as that of the PORTEC-3 trial), or to receive chemotherapy alone (six cycles of carboplatin and paclitaxel).^21^ Although no differences in recurrence-free and overall survival were found, significantly more vaginal recurrences (HR 0·36) and pelvic or para-aortic, or both, recurrences (HR 0·43) were seen in women treated with chemotherapy alone. While using the same chemoradiotherapy schedule as in PORTEC-3, recurrence-free survival in GOG-258 was 59% (97% stage III) compared with 71% in PORTEC-3 for stage III disease. In retrospective cohorts, a high frequency of locoregional recurrence was also found after treatment with chemotherapy alone,^7^, ^8^, ^22^ supporting the continued use of pelvic radiotherapy in patients undergoing adjuvant chemotherapy. Taking the findings of these two recent large trials together, the addition of chemotherapy should be discussed and recommended for women with stage III endometrial cancer to improve failure-free and overall survival as part of shared decision making between doctors and their patients.

Overall and failure-free survival for women with serous cancers were lower than for those with endometrioid and clear cell cancers, and the difference in overall survival and failure-free survival among the different disease stages was more pronounced for women with serous cancers than for those with other histologies. Retrospective studies have reported improvements in overall survival and recurrence-free survival with chemotherapy for serous cancers.^23^, ^24^ In several randomised trials, however, subgroup analyses for treatment effect in different histological types did not confirm such benefits.^19^, ^20^, ^25^ In a GOG study of four randomised chemotherapy trials in women with metastatic and recurrent endometrial cancer, comprising 1203 patients, no association was found between response and histological type.^26^ In the PORTEC-3 trial, however, a significant improvement in both overall survival and failure-free survival was found for women with serous cancers treated with chemoradiotherapy. However, such subgroup analyses should be interpreted with caution for several reasons. The number of patients with serous cancer in these trials is relatively small, and serous cancers have often been grouped together with clear cell cancers. In the current PORTEC-3 analysis, we found that the frequency of recurrence among women with clear cell cancers was similar to that of women with endometrioid tumours and clearly lower than that of women with serous cancers. Another factor to consider when interpreting subgroup analyses is the use of different chemotherapy regimens among the different trials. In the NSGO/EORTC trial,^19^ the GOG-122 trial,^25^ and the GOG metastatic endometrial cancer analysis,^26^ cisplatin-based combinations were used, whereas in the PORTEC-3, GOG-249, and GOG-258 trials, carboplatin and paclitaxel were given.

In the PORTEC-3 trial, 105 patients with serous cancers were included. PORTEC-3 is the first randomised trial to show a significant improvement in overall survival and failure-free survival with combined adjuvant treatment for women with serous cancers, but in absolute terms mostly for higher stage disease. The number of women and events are too low to report on treatment efficacy across the different stages of serous cancers. To achieve further improvements in survival outcomes for women with serous cancers, targeted therapies based on molecular alterations should be explored. Serous cancers are characterised by a high frequency of *TP53* mutations, and in approximately 25% of patients an overexpression of HER2/neu has been reported, with a potential progression-free survival benefit by addition of trastuzumab to chemotherapy.^27^, ^28^

Most first recurrences in the PORTEC-3 trial were at distant sites, with only few simultaneous vaginal or pelvic recurrences. Isolated vaginal or pelvic recurrence was rare. This finding is in line with other randomised trials that used radiotherapy in both groups.^19^, ^20^ In the PORTEC-3 schedule, which was based on the RTOG-9708 phase 2 trial,^12^ both treatment modalities were started early after surgery, aiming at achieving rapid efficacy of both treatment modalities and potential increase of the effect of radiotherapy by the two concurrent cycles. This schedule has now been proven safe and effective in two large, randomised trials with toxicity and quality-of-life data, whereas no phase 3 evidence is available for the specific benefit for other sequences of radiotherapy and chemotherapy.^9^, ^10^, ^19^, ^21^ Widespread differences exist in the practice of giving chemotherapy and radiotherapy in either sequence or in a so-called sandwich schedule; concurrent start of both modalities might be most effective and efficient. Since most first recurrences were at distant sites (although with fewer in the combined treatment group than in the radiotherapy-only group), one could speculate whether multi-agent chemotherapy should already be given concurrently. However, no evidence exists for other concurrent schedules in endometrial cancer, whereas, for example, weekly carboplatin and paclitaxel has been shown to be effective in patients with oesophageal cancer.

In a patient preference study done by the ANZGOG group among their PORTEC-3 participants,^29^ more than 50% of women reported a 5% survival improvement or an extra 1 year survival as being sufficient to make chemotherapy worthwhile. Although both co-primary endpoints showed a significant improvement and are within this range, weighing the benefits of chemoradiotherapy against the increased risk of adverse events remains important. Significantly more severe adverse events and reduced health-related quality of life were reported in the combined treatment group during the first year after treatment.^9^, ^10^ However, rapid recovery was seen, with no significant differences in grade 3–4 adverse events from 12 months onwards. At 60 months after randomisation, significantly more patients reported grade 2 toxicity with the combination treatment, of which sensory neuropathy is the most significant (6% *vs* 0%) and clinically relevant.^9^, ^10^

A limitation of this analysis is that the subgroup analyses were not powered and the survival update was a non-prespecified, post-hoc analysis, with an additional follow-up time of 12 months after 95% of the required failure-free survival events had occurred. However, the previous (time-based) analysis already showed a significant improvement in failure-free survival, and the overall survival difference was close to significance. The differences in overall and failure-free survival have remained and have even become stronger with a longer follow-up time and more events recorded.

In terms of the statistical significance of our findings, the p value for failure-free survival was 0·016 and that for overall survival was 0·034, whereas the boundary for claiming positivity of the study based on Pocock correction was 0·031 (ie, if either or both of the p values were lower than 0·031 the study is deemed positive). The sequential rejection principle^14^ implies that, since the null hypothesis of no treatment difference for failure-free survival was rejected (p<0·031), the null hypothesis of no treatment difference for the co-primary endpoint of overall survival can then be assessed at the 0·05 level, while still retaining a family-wise error rate of 0·05. Application of this principle implies that the treatment effect for overall survival can be considered significant.

Better selection of women for adjuvant treatment might be achieved by integration of molecular characteristics. For intermediate-risk endometrial cancer, an improved risk assessment was reported with an integrated profile of clinicopathological and molecular risk factors.^30^ This approach is currently being tested in the randomised PORTEC-4a trial (NCT03469674). A pilot study of the TransPORTEC consortium showed that determination of the molecular subgroups as described by the Cancer Genome Atlas Group^27^ also improved risk assessment in high-risk endometrial cancer. Translational research is being done on tumour tissue samples donated by PORTEC-3 participants as part of an international collaboration. The molecular subgroups and additional molecular characteristics will be ascertained and the benefit of adjuvant chemotherapy in relation to these factors will be explored.

In conclusion, this updated analysis of the PORTEC-3 trial shows improved 5-year overall and failure-free survival with chemoradiotherapy compared with radiotherapy alone for women with high-risk endometrial cancer, with the greatest absolute benefit for chemotherapy seen in women with stage III disease or serous cancers, or both. Most recurrences were at distant sites, suggesting that new systemic treatment approaches are needed to improve survival outcomes. Molecular analysis has the potential to improve risk stratification and should be used to identify subgroups that can derive the greatest benefit from chemotherapy and to select patients for targeted therapies; molecular studies on tissue samples donated by PORTEC-3 trial participants are ongoing.

**This online publication has been corrected. The corrected version first appeared at thelancet.com/oncology on Sept 2, 2019**

## Data sharing

De-identified participant data will be made available when analyses of all primary and secondary endpoints, including the translational research studies related to the trial, have been published, following the completion of a data transfer agreement. This will specifically be done in an appropriate intergroup setting.

## References

[1] Colombo N, Creutzberg C, Amant F (2015). ESMO–ESGO–ESTRO consensus conference on endometrial cancer: diagnosis, treatment and follow-up. Radiother Oncol.

[2] Straughn JM, Huh WK, Orr JW (2003). Stage IC adenocarcinoma of the endometrium: survival comparisons of surgically staged patients with and without adjuvant radiation therapy. Gynecol Oncol.

[3] Creutzberg CL, van Putten WL, Warlam-Rodenhuis CC (2004). Outcome of high-risk stage IC, grade 3, compared with stage I endometrial carcinoma patients: the Postoperative Radiation Therapy in Endometrial Carcinoma Trial. J Clin Oncol.

[4] Bosse T, Peters EE, Creutzberg CL (2015). Substantial lymph-vascular space invasion (LVSI) is a significant risk factor for recurrence in endometrial cancer—a pooled analysis of PORTEC 1 and 2 trials. Eur J Cancer.

[5] Maggi R, Lissoni A, Spina F (2006). Adjuvant chemotherapy vs radiotherapy in high-risk endometrial carcinoma: results of a randomised trial. Br J Cancer.

[6] Susumu N, Sagae S, Udagawa Y (2008). Randomized phase III trial of pelvic radiotherapy versus cisplatin-based combined chemotherapy in patients with intermediate- and high-risk endometrial cancer: a Japanese Gynecologic Oncology Group study. Gynecol Oncol.

[7] Klopp AH, Jhingran A, Ramondetta L, Lu K, Gershenson DM, Eifel PJ (2009). Node-positive adenocarcinoma of the endometrium: outcome and patterns of recurrence with and without external beam irradiation. Gynecol Oncol.

[8] Mundt AJ, McBride R, Rotmensch J, Waggoner SE, Yamada SD, Connell PP (2001). Significant pelvic recurrence in high-risk pathologic stage I–IV endometrial carcinoma patients after adjuvant chemotherapy alone: implications for adjuvant radiation therapy. Int J Radiat Oncol Biol Phys.

[9] de Boer SM, Powell ME, Mileshkin L (2018). Adjuvant chemoradiotherapy versus radiotherapy alone for women with high-risk endometrial cancer (PORTEC-3): final results of an international, open-label, multicentre, randomised, phase 3 trial. Lancet Oncol.

[10] de Boer SM, Powell ME, Mileshkin L (2016). Toxicity and quality of life after adjuvant chemoradiotherapy versus radiotherapy alone for women with high-risk endometrial cancer (PORTEC-3): an open-label, multicentre, randomised, phase 3 trial. Lancet Oncol.

[11] de Boer SM, Wortman BG, Bosse T (2018). Clinical consequences of upfront pathology review in the randomised PORTEC-3 trial for high-risk endometrial cancer. Ann Oncol.

[12] Greven K, Winter K, Underhill K, Fontenesci J, Cooper J, Burke T (2006). Final analysis of RTOG 9708: adjuvant postoperative irradiation combined with cisplatin/paclitaxel chemotherapy following surgery for patients with high-risk endometrial cancer. Gynecol Oncol.

[13] Pocock SJ, Geller NL, Tsiatis AA (1987). The analysis of multiple endpoints in clinical trials. Biometrics.

[14] Goeman JJ, Solari A (2010). The sequential rejection principle of familywise error control. Ann Statist.

[15] Kahan BC, Morris TP (2012). Reporting and analysis of trials using stratified randomisation in leading medical journals: review and reanalysis. BMJ.

[16] Kahan BC, Morris TP (2012). Improper analysis of trials randomised using stratified blocks or minimisation. Stat Med.

[17] Grambsch PM, Therneau MT (1994). Proportional hazards tests and diagnostics based on weighted residuals. Biometrika.

[18] Putter H, Fiocco M, Geskus RB (2007). Tutorial in biostatistics: competing risks and multi-state models. Stat Med.

[19] Hogberg T, Signorelli M, de Oliveira CF (2010). Sequential adjuvant chemotherapy and radiotherapy in endometrial cancer—results from two randomised studies. Eur J Cancer.

[20] Randall ME, Filiaci V, McMeekin DS (2019). Phase III trial: adjuvant pelvic radiation therapy versus vaginal brachytherapy plus paclitaxel/carboplatin in high-intermediate and high-risk early stage endometrial cancer. J Clin Oncol.

[21] Matei D, Filiaci V, Randall M (2019). Adjuvant chemotherapy plus radiation for locally advanced endometrial cancer. N Engl J Med.

[22] Secord AA, Geller MA, Broadwater G (2013). A multicenter evaluation of adjuvant therapy in women with optimally resected stage IIIC endometrial cancer. Gynecol Oncol.

[23] Viswanathan AN, Macklin EA, Berkowitz R, Matulonis U (2011). The importance of chemotherapy and radiation in uterine papillary serous carcinoma. Gynecol Oncol.

[24] Goldberg H, Miller RC, Abdah-Bortnyak R (2008). Outcome after combined modality treatment for uterine papillary serous carcinoma: a study by the Rare Cancer Network (RCN). Gynecol Oncol.

[25] Randall ME, Filiaci VL, Muss H (2006). Randomized phase III trial of whole-abdominal irradiation versus doxorubicin and cisplatin chemotherapy in advanced endometrial carcinoma: a Gynecologic Oncology Group Study. J Clin Oncol.

[26] McMeekin DS, Filiaci VL, Thigpen JT, Gallion HH, Fleming GF, Rodgers WH (2007). The relationship between histology and outcome in advanced and recurrent endometrial cancer patients participating in first-line chemotherapy trials: a Gynecologic Oncology Group study. Gynecol Oncol.

[27] Kandoth C, Schultz N, Cherniack AD (2013). Integrated genomic characterization of endometrial carcinoma. Nature.

[28] Fader AN, Roque DM, Siegel E (2018). Randomized phase ii trial of carboplatin-paclitaxel versus carboplatin-paclitaxel-trastuzumab in uterine serous carcinomas that overexpress human epidermal growth factor receptor 2/neu. J Clin Oncol.

[29] Blinman P, Mileshkin L, Khaw P (2016). Patients' and clinicians' preferences for adjuvant chemotherapy in endometrial cancer: an ANZGOG substudy of the PORTEC-3 intergroup randomised trial. Br J Cancer.

[30] Stelloo E, Nout RA, Osse EM (2016). Improved risk assessment by integrating molecular and clinicopathological factors in early-stage endometrial cancer-combined analysis of the PORTEC cohorts. Clin Cancer Res.

