# Correction to *Lancet Oncol* 2019; 20: 1273–85

**DOI:** 10.1016/S1470-2045(19)30526-1

**Published:** 2019-09

*de Boer SM, Powell ME, Mileshkin L, et al. Adjuvant chemoradiotherapy versus radiotherapy alone in women with high-risk endometrial cancer (PORTEC-3): patterns of recurrence and post-hoc survival analysis of a randomised phase 3 trial.* Lancet Oncol *2019; **20:** 1273–85*—In this Article, data (hazard ratios, 95% CIs, and p values) in the following sentence on p 1279 have been corrected: “In women with stage I–II disease, 5-year overall survival was 83·8% (95% CI 78·4–89·5) with chemoradiotherapy versus 82·0% (95% CI 76·5–87·7) with radiotherapy alone (HR 0·84 [95% CI 0·52–1·38]; p=0·50), and 5-year failure-free survival was 81·3% (95% CI 74·7–86·3) with chemoradiotherapy versus 77·3% (95% CI 70·5–82·7) with radiotherapy alone (HR 0·87 [95% CI 0·56–1·36] p=0·54; appendix p 8).” On p 1282, the same data (hazard ratios and 95% CIs) in the following sentence have also been corrected: “For women with stage I–II endometrial cancer, combined adjuvant treatment yielded only a small absolute improvement of 2% (HR 0·84; 95% CI 0·52–1·38) in 5-year overall survival and of 4% (0·87; 0·56–1·36) in failure-free survival.” These corrections have been made to the online version as of Sept 2, 2019, and the printed version is correct.

