# Supplementary material for: Adjuvant chemoradiotherapy versus radiotherapy alone in women with high-risk endometrial cancer (PORTEC-3): patterns of recurrence and post-hoc survival analysis of a randomised phase 3 trial
Source: Lancet Oncol. 2019 Sep;20(9):1273–85. doi: 10.1016/S1470-2045(19)30395-X (PMC6722042; doi:10.1016/S1470-2045(19)30395-X)
Supplement: Supplementary appendix [file mmc1.pdf]

THE LANCET
Oncology

Supplementary appendix

This appendix formed part of the original submission and has been peer reviewed. We post it as supplied by the authors.

Supplement to: de Boer S M, Powell M E, Mileshkin L, et al. Adjuvant chemoradiotherapy versus radiotherapy alone in women with high-risk endometrial cancer (PORTEC-3): patterns of recurrence and post-hoc survival analysis of a randomised phase 3 trial. *Lancet Oncol* 2019; published online July 22. http://dx.doi.org/10.1016/S1470-2045(19)30395-X.

PORTEC 3 Study group and participating centres

PORTEC-3 Independent data monitoring committee:
L.V.A.M. Beex, N. James, M.J.M. Olofsen-van Acht, W. Parulekar, W.L.J. van Putten, D. Rischin, J. Yarnold (chair)
PORTEC-3 trial statistician: H. Putter

List of participating countries and centres (listed in order of patients recruited):
United Kingdom: 184 patients (177 evaluable)
Study Coordinators: M. Powell (P.I.), London; H. Kitchener, Manchester; J. Ledermann, London
Group coordinating trial centre: Cancer Research UK and UCL Cancer Trials Centre, London
Trial pathologists: N. Singh, London; G. Wilson, Manchester
Participating centres and principal investigator(s) (number of patients):
London-Barts Health NHS Trust (M. Powell, 23); London-University College London Hospitals NHS Foundation Trust (M. McCormack, 15); Bebington, Wirral-The Clatterbridge Cancer Centre NHS Foundation Trust (K. Whitmarsh, K. Hyatt, 12); Wolverhampton-The Royal Wolverhampton NHS Trust (R. Allerton, 11); Cambridge-Cambridge University Hospitals NHS Foundation Trust (L.T. Tan, M. Iddawela, D. Gregory, S. Ayres, 11); Leicester-Leicester Royal Infirmary (P. Symonds, 10); Northwood-Mount Vernon Cancer Centre (P. Hoskin, 10); Middlesborough-The James Cook University Hospital (A. Rathmell, M. Adusumalli, 9); Nottingham-Nottingham City Hospital (S. Chan, A Anand, 9); Norwich-Norfolk and Norwich University Hospitals NHS Foundation Trust (R. Wade, 8); Guildford-Royal Surrey County Hospital (A. Stewart, 6); Newcastle Upon Tyne-Freeman Hospital (W. Taylor, 6); Brighton-Brighton & Sussex University Hospitals NHS Trust (K. Lankester, 5); Coventry-University Hospital Coventry (C. Irwin, M. Hocking, 5); Taunton-Musgrove Park Hospital (P. Jankowska, D. Milliken, C. Barlow, 5); Cheltenham-Cheltenham General Hospital (A. Cook, R. Counsell, 4); Exeter-Royal Devon & Exeter Hospital (P. Bliss, A. Hong, 4); Lincoln-Lincoln County Hospital (M. Panades, 4); Romford-Queens Hospital (M. Quigley, 4); Manchester-The Christie NHS Foundtion Trust (S. Davidson, 3); Northampton-Northampton General Hospital NHS Trust (C. Mak, 3); Preston-Royal Preston Hospital (A. Hindley, 3); Truro-Royal Cornwall Hospitals NHS Trust (A. Thomson, 3); Sheffield-Weston Park Hospital (S. Pledge, J. Martin, 2); Shrewsbury-Royal Shrewsbury Hospital (S. Awwad, A. Zachariah, 2); Carlisle-North Cumbria University Hospitals NHS Trust (S. Singhal, 1); Colchester-Essex County Hospital (A. Lamont, 1); London-Guy's and St. Thomas' NHS Foundation Trust (A. Winship, A. Montes, V. Mullassery, 1); London-Hammersmith Hospital - Imperial College Healthcare NHS Trust (A. Taylor, 1); Poole-Poole Hospital NHS Foundation Trust (V. Laurence, M. Flubacher, 1); Reading-Royal Berkshire Hospital (H. O'Donnell, 1); Stoke-on-Trent-Royal Stoke University Hospital (R. Bhana, S. Lupton, 1)

The Netherlands: 145 patients (138 evaluable)
Study Coordinators: C.L. Creutzberg (C.I.), Leiden; R. Kruitwagen, Maastricht; H. Nijman, Groningen; N. Ottevanger, Nijmegen
Group coordinating trial centre: Netherlands Comprehensive Cancer Organisation (IKNL), Leiden
Trial pathologists: H. Hollema, Groningen; V.T. Smit, Leiden
Participating centres and principal investigator(s) (number of patients):
University Medical Center Utrecht (I.M. Jurgenliemk-Schulz, 20); Maastro Clinic, Maastricht (L.C.H.W. Lutgens, 17); University Medical Center Groningen (E. Pras, 15); Leiden University Medical Center (C.L. Creutzberg, R. Nout, 15); University Medical Center Radboud, Nijmegen (J.W.H. Leer, A. Snyers, 11); Academic Medical Center, Amsterdam (A.L.J. Uitterhoeve, G.H. Westerveld, 9); Medical Spectre Twente, Enschede (J.J. Jobsen, 9); Radiotherapy institute Friesland, Leeuwarden (A. Slot, 9); Erasmus Medical Center Rotterdam (J.W.M. Mens, 8); Medical Center Haaglanden/ Radiotherapy Centre West (T.C. Stam, P.C.M. Koper, 7); Netherlands Cancer Institute, Amsterdam (B. van Triest, 6); Radiotherapy Group, Arnhem (E.M. van der Steen-Banasik, 6); Radiotherapy Institute Verbeeten, Tilburg (K.A.J. de Winter, 6); Radiotherapy Group, Deventer (S. van de Pol, 3); Catharina Hospital, Eindhoven (H.A. van den Berg, 3); VU Medical Centre, Amsterdam (O.W.M. Meijer, 1)

Australia & New Zealand: 122 patients (118 evaluable)
ANZGOG Study Coordinators: L. Mileshkin (P.I.) Melbourne; M. Quinn, Melbourne; P. Khaw Melbourne; I.Kolodziej, Sydney

Group coordinating trial centre: NHMRC Clinical Trials Centre, Sydney
Trial pathologist: J.Pyman, Melbourne
Participating centres and principal investigator(s) (number of patients):
Peter MacCallum Cancer Centre, Melbourne, Victoria, Australia (L. Mileshkin, P.Khaw; 31); Monash Cancer Centre (Monash Medical Centre), East Bentleigh, Victoria, Australia (P. Khaw/G. Goss, 20); Westmead Hospital, Wentworthville, NSW, Australia (G. Wain, 15); Auckland City Hospital , Auckland, New Zealand (S. Brooks, 13); Wellington Blood & Cancer Centre, Wellington, New Zealand (C. Johnson, 11); Calvary Mater Newcastle, Newcastle, Australia (A. Capp, 8); Christchurch Hospital, Canterbury, New Zealand (M. Vaughan, 4); Royal Hobart Hospital, Hobart, Tasmania, Australia (P. Blomfield, 3); Palmerston North Hospital, Palmerston North, New Zealand (C. Hardie, 3); Royal North Shore Hospital, St Leonards, NSW, Australia (M. Stevens, 3); Waikato Hospital, Hamilton, New Zealand (M. Kuper, 2); Royal Brisbane &Women's Hospital, Brisbane, QLD, Australia(R. Cheuk, 2); Liverpool Hospital, Liverpool, NSW, Australia (S. Vinod, 2); Mater Hospital Brisbane , South Brisbane, QLD, Australia(C. Shannon/J. Ramsay, 2); Wollongong Hospital, Wollongong, NSW, Australia (A. Glasgow, 2); Townsville Hospital, Townsville, QLD, Australia (S. Hewitt, 1)

Italy: 103 patients (98 evaluable)
MaNGO Study Coordinators: R. Fossati (P.I.) Milano; D. Katsaros, Torino; A. Colombo, Lecco
Group coordinating trial centre: Istituto di Ricerche Farmacologiche Mario Negri, Milano
Trial pathologists: S. Carinelli, Milano; C. Di Tonno, Milano
Participating centres and principal investigator(s) (number of patients):
Torino - S. Anna Hospital (S. Gribaudo, M. Mitidieri, 33); Lecco - Ospedale A. Manzoni (R. D'Amico, 24); Monza - S. Gerardo Hospital (S. Meregalli, A. A. Lissoni, 16); Torino - Ospedale Umberto I (A. Ferrero, 7); Padova – Istituto Oncologico Veneto / Mirano, Venezia – Azienda ULSS 13 (L. Corti, G. Artioli, 6); Varese - H. Del Ponte University of Insubria (C. Apolloni, 4); Ravenna - Ospedale S. Maria delle Croci (D. Turci, 4); Brescia – Spedali Civili (G. Tognon, 2); Como – ASST Lariana Ospedale S. Anna (E. Bianchi, 2); Meldola - Istituto Scientifico Romagnolo per lo Studio e la Cura dei Tumori (E. Bianchi, 2); Genova – IRCCS San Martino IST (M. Bruzzone, 1); Milano - ASST Grande Ospedale Metropolitano Niguarda (S. Siena, 1); Palermo – AOR Villa Sofia-Cervello (N. Varsellona, 1)

Canada: 65 patients (65 evaluable)
CCTG Study Coordinators: A. Fyles Toronto, Ontario; P. Bessette, Sherbrooke, Quebec
Group coordinating trial centre: Canadian Cancer Trials Group, Kingston, Ontario
Trial pathologist: M. McLachlin, London, Ontario
Participating centres and principal investigator(s) (number of patients):
Sherbrooke-Centre Hosp. Universitaire de Sherbrooke (P. Bessette, 18); Montreal-Hopital Notre-Dame de Montreal (D. Provencher, 11); Calgary-Tom Baker Cancer Centre (P. Ghatage, 9); Halifax-Queen Elizabeth II Health Sciences Centr (P. Rittenberg, 8); Montreal-McGill Oncology Montreal (L. Souhami, 7); Toronto-Sunnybrook Health Sciences Centre (G. Thomas, 7); Quebec-Hotel-Dieu de Quebec (M. Plante, 2); London-London Health Sciences Centre (A. Hammond, 1); St John's-Dr. H. Bliss Murphy Cancer Centre (P. Power, 1); Toronto-Princess Margaret Hospital (A. Fyles, 1)

France: 67 patients (64 evaluable)
FEDEGYN Study Coordinator: Chr. Haie-Meder (P.I.) Paris
Group coordinating trial centre: UNICANCER, Paris
Trial pathologist: P. Duvillard, Paris
Participating centres and principal investigator(s) (number of patients):
Besancon-Hopital Jean Minjoz (M-H Baron, 10); Rouen-Centre Henri Becquerel (Hanzen, 9); Saint Herblain-Centre Rene Gauducheau (D. Berton-Rigaud, 8); Limoges-CHU Limoges (Pr. N. Tubiana-Mathieu, 6); Bordeaux-Institut Bergonie (L. Thomas, 5); Reims-Institut Jean Godinot (A. Savoye, S. Maillard, 5); Dijon-Centre Georges Francois Leclerc (K. Peignaux, 4); Paris/ Villejuif-Institut Gustave Roussy (C. Haie Meder, 4); Clermont-Ferrand-Centre Jean Perrin (C. Benoit, 3); Montpellier-Centre Val d'Aurelle (C. Kerr, 3); Toulouse Cedex-Institut Claudius Regaud (L. Gladieff, 3); Caen-Centre Francois Baclesse (D. Lerouge, 2); Nice Cedex-Centre Antoine Lacassagne (P. Follana, 2); Marseille-Institut Paoli Calmettes (M. Cappiello, 1); Strasbourg-Centre Paul Strauss (T. Petit, 1); Tours Cedex-CHU de Tours - Hopital Bretonneau (I. Barillot, 1)

Table S1: Treatment for first recurrence

	Total		RT		RT + CT	
**Treatment**	**N**	**%**	**N**	**%**	**N**	**%**
Chemotherapy*	78	42·2%	48	46·6%	30	36·6%
Hormonal therapy	26	14·1%	13	12·6%	13	15·9%
Radiotherapy	15	8·1%	7	6·8%	8	9·8%
Surgery	11	5·9%	7	6·8%	4	4·9%
Targeted therapy	2	1·1%	0	0·0%	2	2·4%
Surgery + radiotherapy	5	2·7%	1	1·0%	4	4·9%
Surgery + hormonal therapy	2	1·1%	0	0·0%	2	2·4%
Surgery + chemotherapy	4	2·2%	3	2·9%	1	1·2%
Surgery + radiotherapy + chemotherapy	1	0·5%	1	1·0%	0	0·0%
Radiotherapy + hormonal therapy	6	3·2%	3	2·9%	3	3·7%
Radiotherapy + chemotherapy	5	2·7%	2	1·9%	3	3·7%
Chemotherapy + hormonal therapy	2	1·1%	2	1·9%	0	0·0%
Unknown	1	0·5%	1	1·0%	0	0·0%
No treatment	27	14·6%	15	14·6%	12	14·6%
Total	185	100·0%	103	100·0%	82	100·0%

* Radiotherapy group: 43 patients received carboplatin + paclitaxel; 2 carboplatin; 1 adriamycine + cisplatin; 1 paclitaxel; 1 paclitaxel, epirubicine, cisplatin). Chemoradiotherapy group: 12 patients received carboplatin + paclitaxel; 5 adriamycine + cisplatin; 4 carboplatin + doxorubicine; 3 doxorubicine; 2 carboplatin + epirubucine; 1 paclitaxel; 1 cyclophosphamide, adriamycine, cisplatin; 1 apebilone; 1 unknown.

Abbreviations: CTRT: chemotherapy plus radiotherapy. RT: radiotherapy.

Table S2: Late toxicity reported by physicians using the CTCAE v3.0 at 12, 36 and 60 months of follow-up.

	12 months CTRT n=316; RT n=319						36 months CTRT n=272; RT n=273						60 months CTRT n=201; RT n=187					
	Grade 2			Grade 3/4			Grade 2			Grade 3/4			Grade 2			Grade 3/4		
	CTRT n (%)	RT n (%)	p*	CTRT n (%)	RT n (%)	p#	CTRT n (%)	RT n (%)	p*	CTRT n (%)	RT n (%)	p#	CTRT n (%)	RT n (%)	p*	CTRT n (%)	RT n (%)	p#
**Any**	103 (33)	89 (28)	0·04	33 (10)	21 (7)	0·09	62 (23)	49 (18)	0·06	22 (8)	16 (6)	0·32	59 (29)	33 (18)	0·002	17 (8)	10 (5)	0·24
**Any grade 3**	na	na		27 (9)	19 (6)		na	na		21 (8)	17 (7)		na	na		16 (8)	10 (5)	
**Any grade 4**	na	na		6 (2)	2 (1)		na	na		1 (0)	0 (0)		na	na		1 (0)	0 (0)	
Auditory/hearing	10 (3)	2 (1)	0·02	0 (0)	0 (0)	1·00	1 (0)	1 (0)	1·00	1 (0)	1 (0)	1·00	4 (2)	1 (1)	0·29	2 (1)	1 (1)	1·00
Hypertension	14 (4)	17 (5)	0·62	4 (1)	5 (2)	1·00	15 (6)	17 (6)	0·75	5 (2)	6 (2)	1·00	16 (8)	16 (9)	0·87	4 (2)	4 (2)	1·00
Lymphatics (edema)	8 (3)	3 (1)	0·11	2 (1)	1 (0)	0·62	3 (1)	1 (0)	0·12	2 (1)	0 (0)	0·25	5 (2)	0 (0)	0·06	0 (0)	0 (0)	1·00
**GI -** any	21 (7)	19 (6)	0·24	7 (2)	2 (1)	0·11	11 (4)	17 (6)	0·46	2 (1)	1 (0)	0·62	16 (8)	9 (5)	0·19	2 (1)	1 (1)	1·00
Diarrhea	11 (3)	8 (3)	0·39	1 (0)	1 (0)	1·00	4 (1)	8 (3)	0·42	1 (0)	1 (0)	1·00	7 (3)	7 (4)	1·00	0 (0)	0 (0)	1·00
Ileus/obstruction	2 (1)	3 (1)	0·77	4 (1)	2 (1)	0·45	0 (0)	0 (0)	0·50	1 (0)	0 (0)	0·50	2 (1)	1 (1)	0·37	2 (1)	0 (0)	0·50
**Hematological** - any	26 (8)	21 (7)	0·89	4 (1)	7 (2)	0·55	3 (1)	3 (1)	1·00	1 (0)	2 (1)	1·00	5 (2)	5 (3)	1·00	0 (0)	0 (0)	1·00
Lymphocytes	24 (8)	22 (7)	0·89	4 (1)	5 (2)	1·00	3 (1)	3 (1)	1·00	2 (1)	1 (0)	1·00	3 (1)	4 (2)	0·74	0 (0)	0 (0)	1·00
**Neuropathy -** any	26 (8)	1 (0)	<0·001	4 (1)	1 (0)	0·22	17 (6)	2 (1)	<0·001	3 (1)	0 (0)	0·12	13 (6)	0 (0)	<0·001	1 (0)	0 (0)	1·01
Neuropathy - motor	1 (0)	0 (0)	0·22	3 (1)	1 (0)	0·37	3 (1)	2 (1)	0·45	1 (0)	0 (0)	0·50	1 (0)	0 (0)	0·50	1 (0)	0 (0)	1·02
Neuropathy - sensory	26 (8)	1 (0)	<0·001	4 (1)	1 (0)	0·22	17 (6)	1 (0)	<0·001	3 (1)	0 (0)	0·12	12 (6)	0 (0)	<0·001	1 (0)	0 (0)	1·03
**Pain** - any	26 (8)	22 (7)	0·18	8 (3)	3 (1)	0·14	17 (6)	15 (5)	0·24	4 (1)	0 (0)	0·06	13 (6)	5 (3)	0·15	3 (1)	3 (2)	1·00
Joint pain	4 (1)	3 (1)	0·72	0 (0)	0 (0)	1·00	2 (1)	5 (2)	0·72	1 (0)	0 (0)	0·50	7 (3)	2 (1)	0·14	2 (1)	1 (1)	1·00
Muscle pain	4 (1)	1 (0)	0·22	0 (0)	0 (0)	1·00	0 (0)	3 (1)	0·25	0 (0)	0 (0)	1·00	1 (0)	1 (1)	0·61	0 (0)	1 (1)	0·48
Back/pelvic/limbs	11 (3)	5 (2)	0·05	3 (1)	1 (0)	0·37	4 (1)	3 (1)	0·50	1 (0)	0 (0)	0·50	0 (0)	2 (1)	0·11	0 (0)	1 (1)	0·48
abdomen/cramps	2 (1)	5 (2)	0·77	3 (1)	2 (1)	0·69	4 (1)	1 (0)	0·12	1 (0)	0 (0)	0·50	2 (1)	0 (0)	0·12	2 (1)	0 (0)	0·50
**Musculoskeletal** (other)	3 (1)	0 (0)	0·12	0 (0)	0 (0)	1·00	1 (0)	0 (0)	0·62	1 (0)	0 (0)	0·50	0 (0)	1 (1)	1·00	1 (0)	0 (0)	1·00
**Pulmonary** - dyspnea	2 (1)	2 (1)	1·00	0 (0)	1 (0)	1·00	1 (0)	0 (0)	1·00	0 (0)	1 (0)	1·00	2 (1)	0 (0)	0·25	0 (0)	0 (0)	1·00
**Genitourinary**																		
Incontinence	8 (3)	9 (3)	1·00	1 (0)	1 (0)	1·00	8 (3)	3 (1)	0·09	1 (0)	0 (0)	0·50	8 (4)	9 (5)	1·00	0 (0)	0 (0)	1·00
Obstruction	0 (0)	0 (0)	1·00	0 (0)	1 (0)	1·00	0 (0)	0 (0)	1·00	0 (0)	1 (0)	1·00	0 (0)	0 (0)	1·00	0 (0)	0 (0)	1·00
Urinary frequency	4 (1)	8 (3)	0·26	0 (0)	1 (0)	1·00	7 (3)	5 (2)	0·58	0 (0)	0 (0)	1·00	8 (4)	2 (1)	0·14	0 (0)	1 (1)	0·48
**Constitutional**																		
Fatigue	5 (2)	4 (1)	1·00	0 (0)	2 (1)	0·50	1 (0)	0 (0)	0·50	0 (0)	0 (0)	1·00	0 (0)	3 (2)	0·11	0 (0)	0 (0)	1·00
Other	5 (2)	4 (1)	0·54	1 (0)	0 (0)	0·50	1 (0)	0 (0)	0·25	1 (0)	0 (0)	0·50	0 (0)	0 (0)	1·00	1 (0)	0 (0)	1·00
Other toxicity	0 (0)	0 (0)	1·00	0 (0)	0 (0)	1·00	2 (1)	3 (1)	1·00	1 (0)	1 (0)	1·00	4 (2)	4 (2)	1·00	3 (1)	2 (1)	1·00

Abbreviations: CTRT, combined chemotherapy and radiotherapy; RT, radiotherapy; GI, gastro-intestinal; GU, genito-urinary; CTCAE v3.0, Common Terminology Criteria for Adverse Events version 3.0; AE were calculated at each time point. Per AE, the maximum grade per patient was calculated (worst ever by patient). p* = significant level < 0.01 for grade ≥2, 3 and 4; p# = significant level <0.01 for grade 3 and 4.

Table S3: Multivariable analysis of prognostic factors for overall survival and failure-free survival

Overall Survival					
	# of patients	# of events	5yrs OS (95% CI)	HR (95% CI)	P-value
**Total**	660	150			
**Prognostic variables**					
**Arm**					0·022
Radiotherapy (RT)	330	85	76% (71·6-80·9)		
Chemoradiotherapy (CTRT)	330	65	81% (77·2-85·8)	0·68 (0·49 - 0·95)	
**Age**					<0·001
< 60 years	268	33	89% (85·2-92·8)		
60-69 years	272	73	75% (69·6-80·1)	2·35 (1·54-3·58)	
≥70 years	120	44	65% (57·1-74·5)	3·42 (2·12-5·52)	
**Stage**					<0·001
Stage I + II	365	66	83% (79·0-86·9)		
Stage III	295	84	74% (68·7-78·9)	2·50 (1·75 - 3·56)	
**Histology and grade**					<0·001
Endometrioid grade 1 + 2	258	39	86% (82·2-90·7)		
Endometrioid grade 3	213	49	78% (72·9-84·2)	1·91 (1·22 - 2·98)	
Serous / clear cell	189	62	69% (62·4-75·8)	2·57 (1·66 - 3·97)	
**LVSI**					0·162
No	271	50	83% (78·7-87·9)		
Yes	389	100	76% (71·4-80·1)	1·29 (0·90 - 1·84)	
**Lymphadenectomy**					0·119
No	281	71	75% (70·5-80·8)		
Yes	379	79	81% (77·3-85·3)	0·74 (0·51 - 1·08)	

* Adjusted for participating groups

Failure-Free survival					
	# of patients	# of events	5yrs FFS	HR (95% CI)	P-value
**Total**	660	189			
**Prognostic variables**					
**Arm**					0·007
Radiotherapy (RT)	330	105	69% (63·8-73·8)		
Chemoradiotherapy (CTRT)	330	84	76% (71·5-80·7)	0·67 (0·50-0·90)	
**Age**					<0·001
< 60 years	268	54	81% (75·9-85·4)		
60-69 years	272	89	68% (62·0-73·2)	1·78 (1·26 - 2·52)	
≥70 years	120	46	65% (55·7-72·7)	2·20 (1·45 - 3·32)	
**Stage**					<0·001
Stage I + II	365	79	79% (74·6-83·1)		
Stage III	295	110	65% (59·0-70·0)	2·62 (1·90-3·60)	
**Histology and grade**					<0·001
Endometrioid grade 1 + 2	258	61	79% (73·6-83·6)		
Endometrioid grade 3	213	60	73% (66·0-78·1)	1·54 (1·05-2·26)	
Serous / clear cell	189	68	64% (57·1-70·8)	2·12 (1·45-3·10)	
**LVSI**					0·042
No	271	63	78% (72·8-82·7)		
Yes	389	126	69% (64·1-73·3)	1·38 (1·01-1·89)	
**Lymphadenectomy**					0·227
No	281	84	72% (65·9-76·6)		
Yes	379	105	74% (68·8-77·8)	0·81 (0·58-1·14)	

* Adjusted for participating groups

Abbreviations: CTRT: chemotherapy plus radiotherapy. RT: radiotherapy. LVSI: lymph vascular space invasion. HR: hazard ratio. CI: confidence interval. OS: overall survival. FFS: failure-free survival.

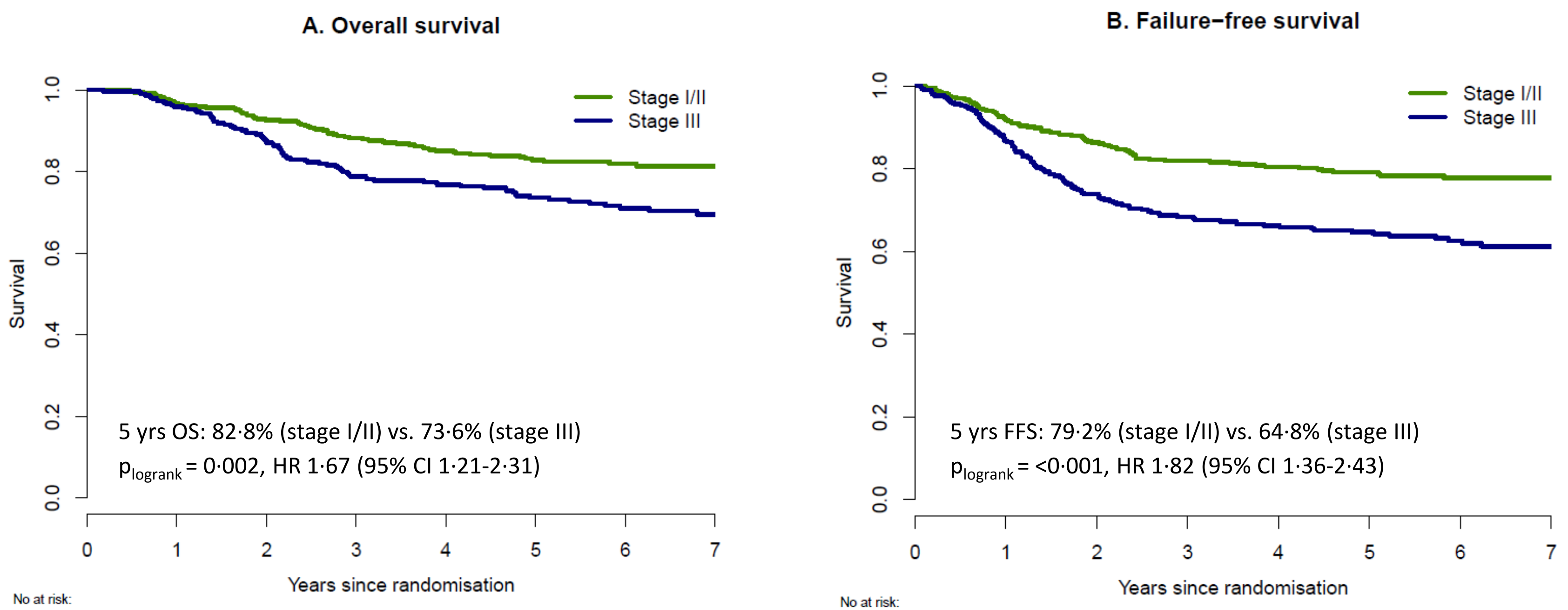

A. No at risk:

	0	1	2	3	4	5	6	7
Stage I/II:	365	353	338	317	290	217	146	99
Stage III:	295	282	256	228	216	171	120	71

A. No censored:

	0	1	2	3	4	5	6	7
Stage I/II:	0	0	0	5	21	87	156	202
Stage III:	0	1	2	5	11	48	94	141

B. No at risk:

	0	1	2	3	4	5	6	7
Stage I/II:	365	335	315	290	269	201	133	92
Stage III:	295	255	217	196	183	144	103	61

B. No censored:

	0	1	2	3	4	5	6	7
Stage I/II:	0	0	0	9	25	89	154	195
Stage III:	0	1	1	6	13	48	85	125

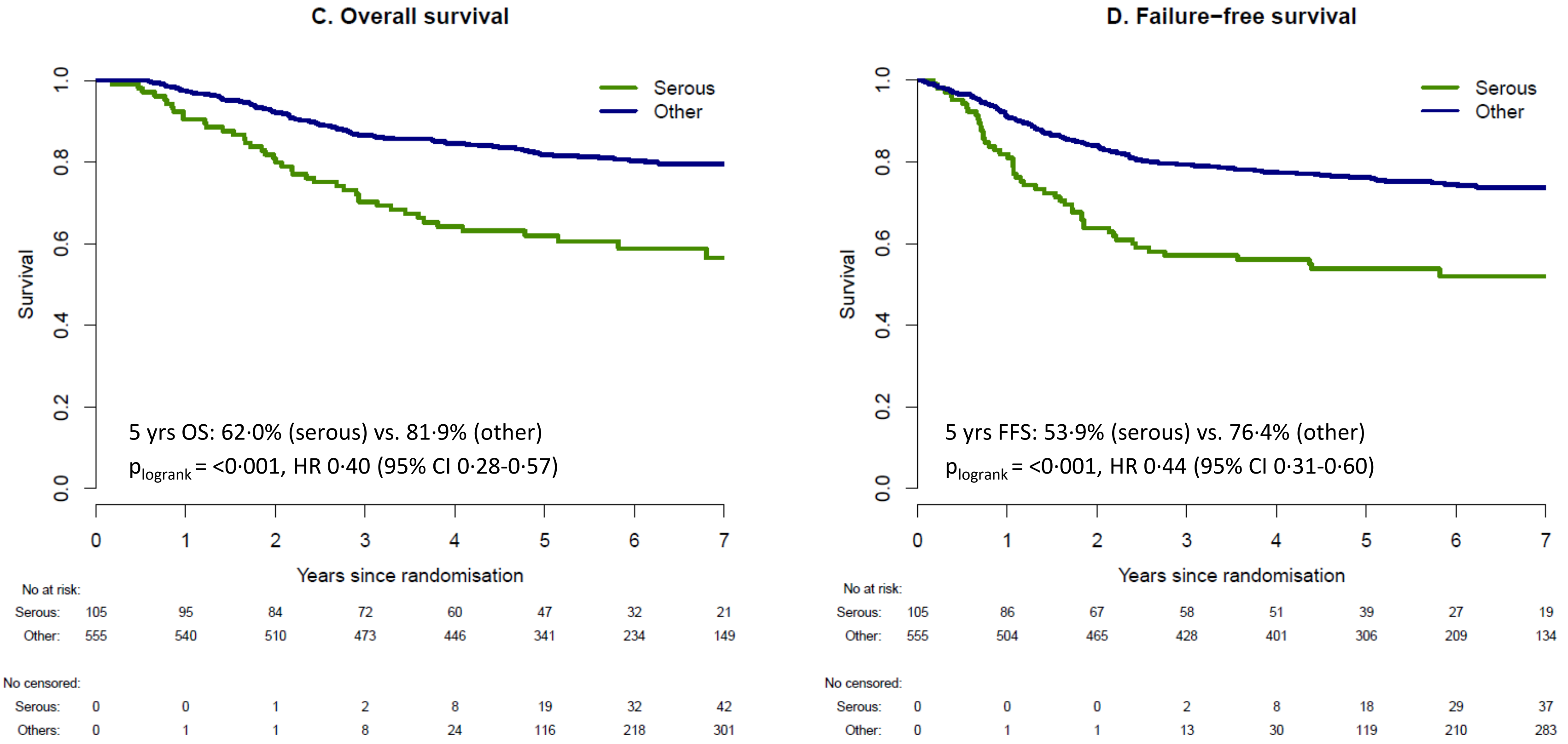

Figure S4: Overall and failure-free survival

Kaplan-Meier survival curve for overall survival (A) and failure free survival (B) in patients with stage I/II versus stage III endometrial cancer. Overall survival and failure-free survival for patients with serous cancer versus all other histological types (C and D).

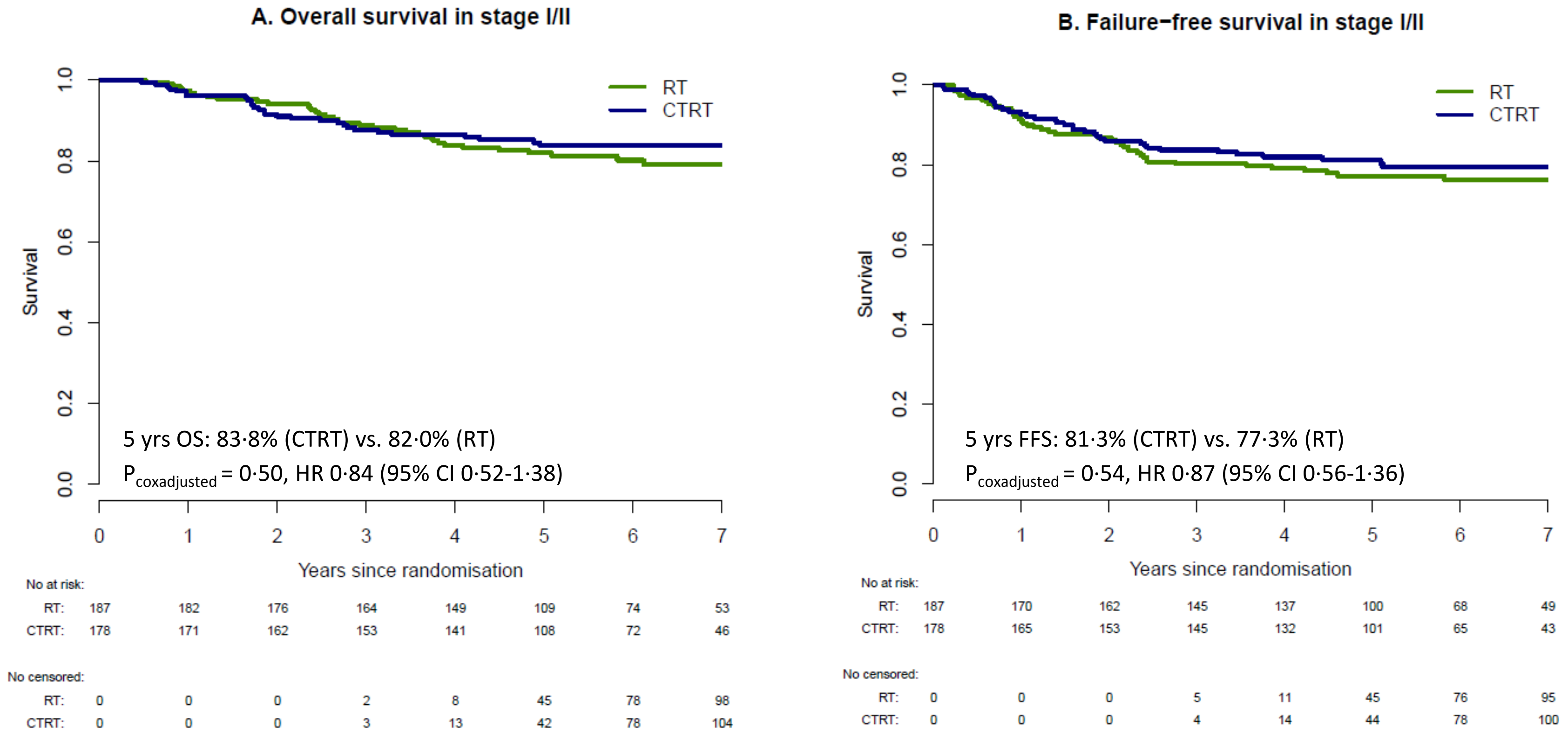
A. Overall survival in stage I/II
RT
CTRT
Survival
0.0
0.2
0.4
0.6
0.8
1.0
5 yrs OS: 83·8% (CTRT) vs. 82·0% (RT)
$P_{coxadjusted}$ = 0·50, HR 0·84 (95% CI 0·52-1·38)
0 1 2 3 4 5 6 7
Years since randomisation
No at risk:
RT: 187 182 176 164 149 109 74 53
CTRT: 178 171 162 153 141 108 72 46
No censored:
RT: 0 0 0 2 8 45 78 98
CTRT: 0 0 0 3 13 42 78 104
B. Failure−free survival in stage I/II
RT
CTRT
Survival
0.0
0.2
0.4
0.6
0.8
1.0
5 yrs FFS: 81·3% (CTRT) vs. 77·3% (RT)
$P_{coxadjusted}$ = 0·54, HR 0·87 (95% CI 0·56-1·36)
0 1 2 3 4 5 6 7
Years since randomisation
No at risk:
RT: 187 170 162 145 137 100 68 49
CTRT: 178 165 153 145 132 101 65 43
No censored:
RT: 0 0 0 5 11 45 76 95
CTRT: 0 0 0 4 14 44 78 100

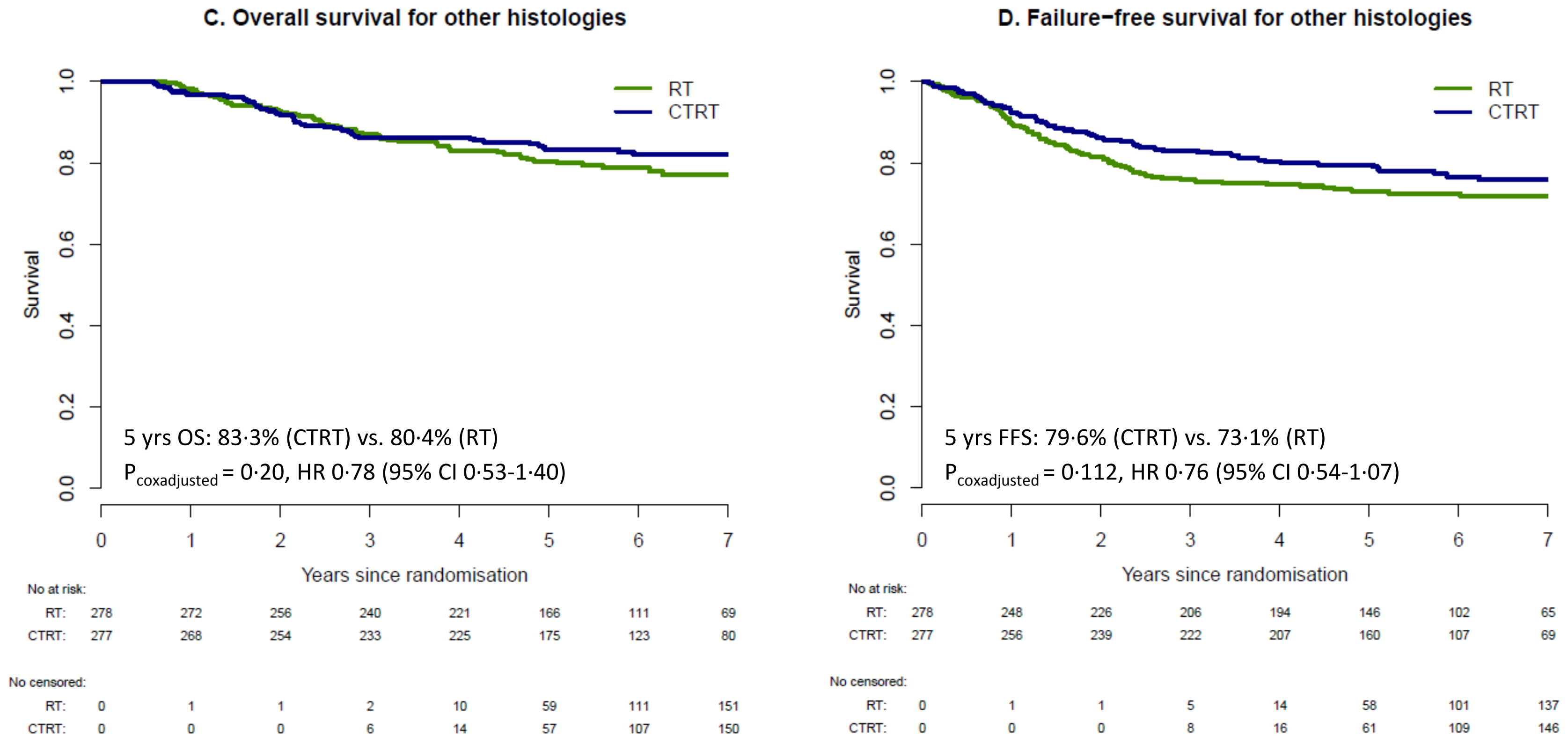

Figure S5: Overall and failure-free survival

Kaplan-Meier survival curve for overall survival (A) and failure free survival (B) in patients with stage I/II endometrial cancer. Overall survival and failure-free survival of patients with a histological type other than serous type (C and D).

A. Overall survival

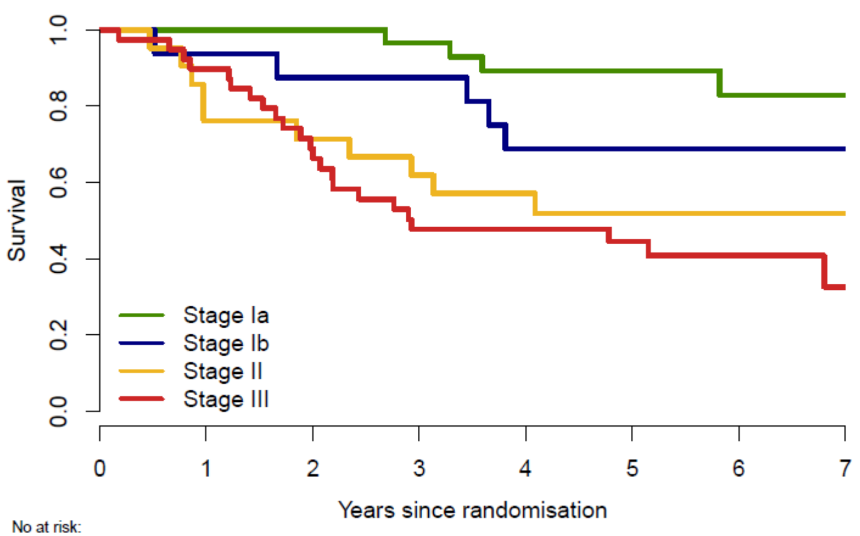

No at risk:

	0	1	2	3	4	5	6	7
Stage Ia:	29	29	29	27	21	16	12	11
Stage Ib:	16	15	14	14	11	11	6	5
Stage II:	21	16	15	13	11	7	5	3
Stage III:	39	35	26	18	17	13	9	2

No censored:

	0	1	2	3	4	5	6	7
Stage Ia:	0	0	0	1	5	10	13	14
Stage Ib:	0	0	0	0	0	0	5	6
Stage II:	0	0	0	0	1	4	6	8
Stage III:	0	0	1	1	2	5	8	14

B. Failure−free survival

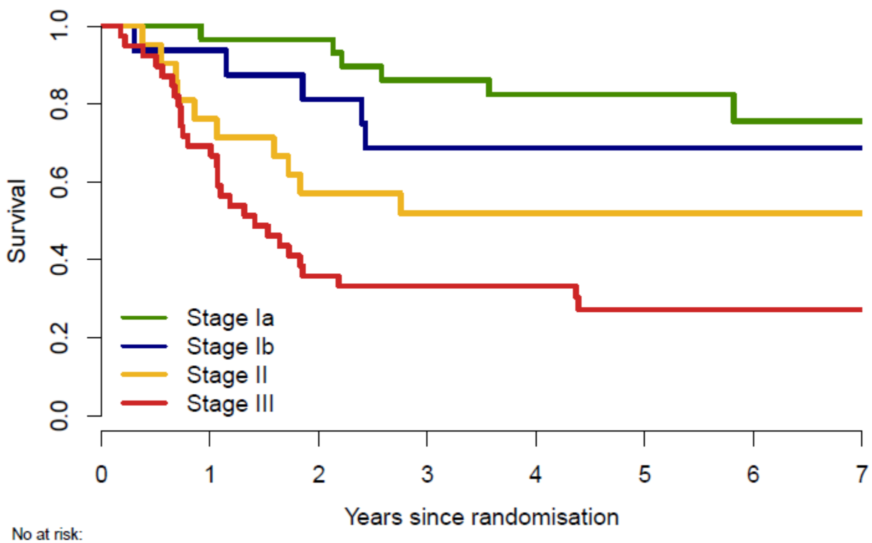

No at risk:

	0	1	2	3	4	5	6	7
Stage Ia:	29	28	28	24	19	14	10	9
Stage Ib:	16	15	13	11	11	11	6	5
Stage II:	21	16	12	10	9	6	5	3
Stage III:	39	27	14	13	12	8	6	2

No censored:

	0	1	2	3	4	5	6	7
Stage Ia:	0	0	0	1	5	10	13	14
Stage Ib:	0	0	0	0	0	0	5	6
Stage II:	0	0	0	1	2	5	6	8
Stage III:	0	0	0	0	1	3	5	9

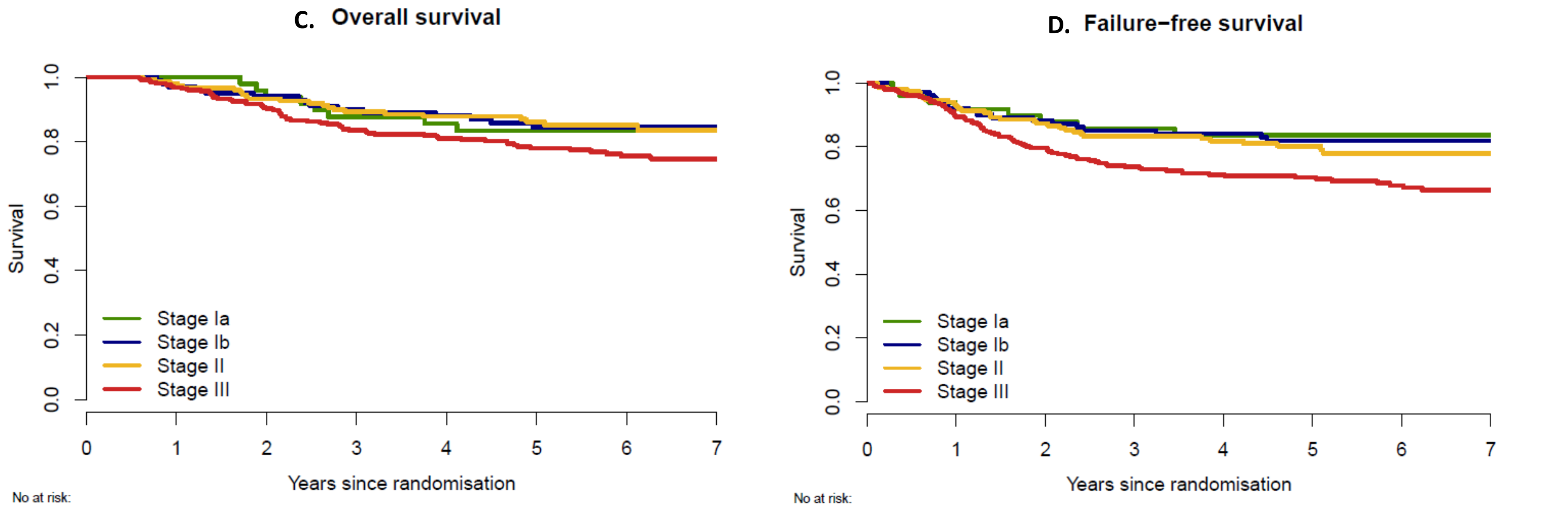

C. Overall survival

No at risk:

	0	1	2	3	4	5	6	7
Stage Ia:	49	49	46	43	40	30	26	14
Stage Ib:	101	98	95	89	86	60	39	24
Stage II:	149	146	139	131	121	93	58	42
Stage III:	256	247	230	210	199	158	111	69

No censored:

	0	1	2	3	4	5	6	7
Stage Ia:	0	0	0	0	2	11	15	27
Stage Ib:	0	0	0	2	3	26	47	62
Stage II:	0	0	0	2	10	36	70	85
Stage III:	0	1	1	4	9	43	86	127

D. Failure−free survival

No at risk:

	0	1	2	3	4	5	6	7
Stage Ia:	49	45	43	41	38	29	25	13
Stage Ib:	101	93	89	84	82	57	36	23
Stage II:	149	138	130	120	110	84	51	39
Stage III:	256	228	203	183	171	136	97	59

No censored:

	0	1	2	3	4	5	6	7
Stage Ia:	0	0	0	1	3	12	16	28
Stage Ib:	0	0	0	2	3	26	47	60
Stage II:	0	0	0	4	12	36	67	79
Stage III:	0	1	1	6	12	45	80	116

Figure S6: Overall and failure-free survival

Kaplan-Meier survival curves for overall survival (A) and failure free survival (B) in patients with serous endometrial cancer among the different stages; and overall survival (C) and failure-free survival (D) in patients with a histological type other than serous cancer among different stages.

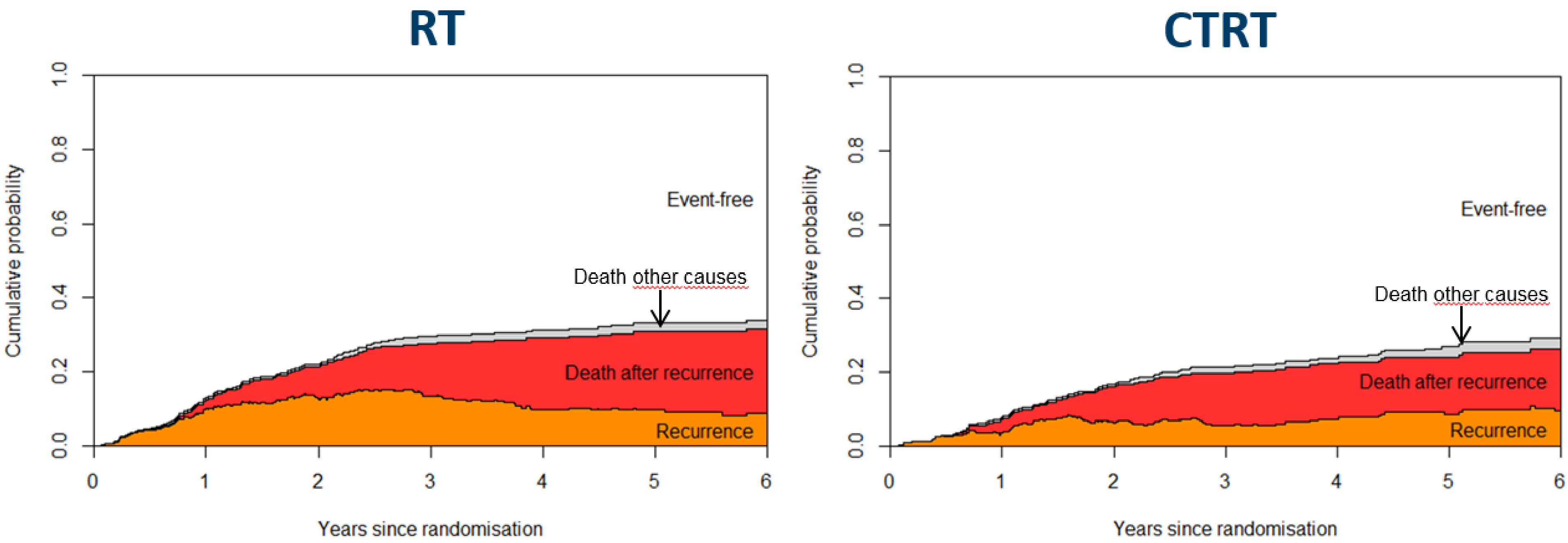

Figure S7: Events and survival by arm
For every time point since randomisation the cumulative probability for a patient was given to be: event-free, alive with an event, deceased after event or deceased due to other causes (intercurrent or second cancer).

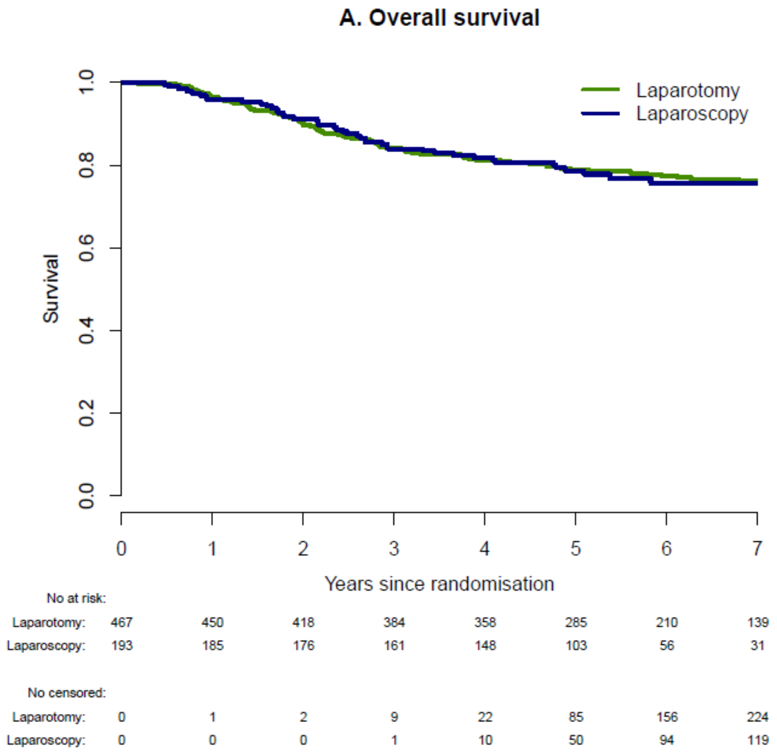

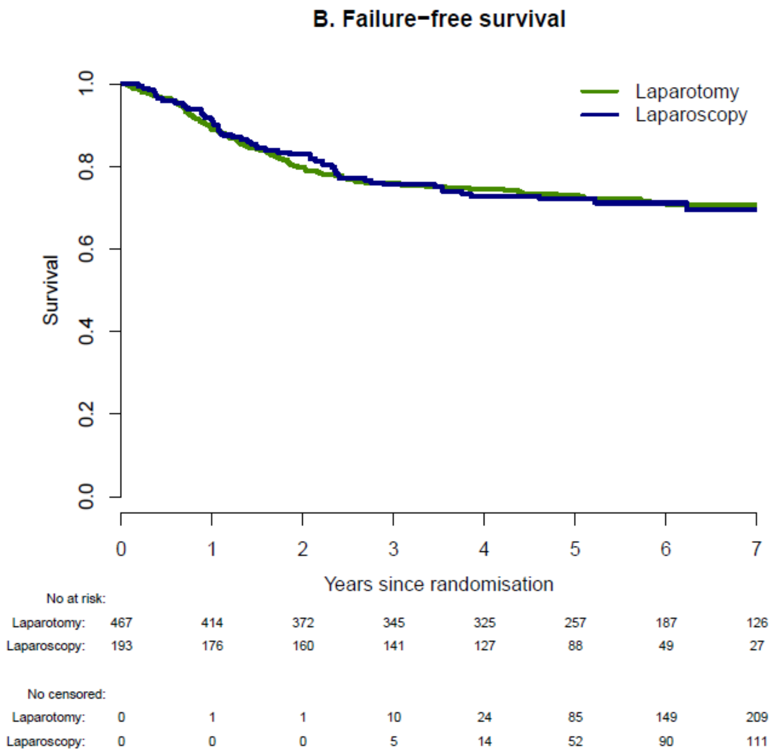

Figure S8: Overall and failure-free survival

Kaplan-Meier survival curve for overall survival (A) and failure free survival (B) in patients who underwent laparotomy compared with patient who underwent laparoscopic surgery

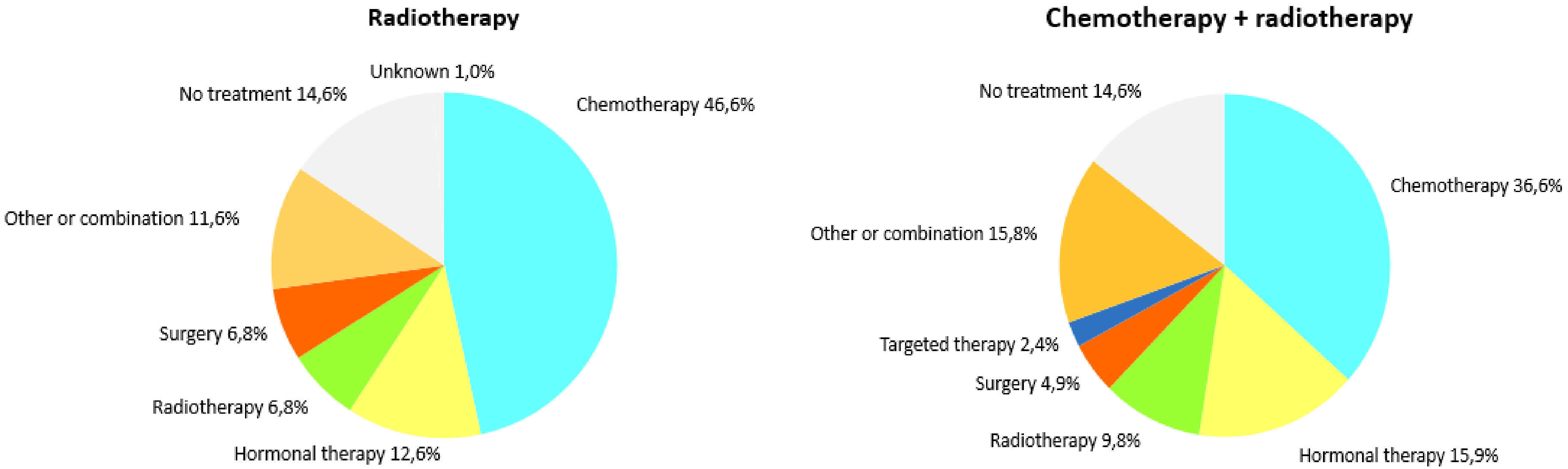

Figure S9: Treatment for first recurrences by treatment arm
